# The improvement of block chain technology simulation in supply chain management (case study: pesticide company)

**DOI:** 10.1038/s41598-024-53694-w

**Published:** 2024-02-15

**Authors:** Lina Gozali, Helena Juliana Kristina, Andrew Yosua, Teuku Yuri M. Zagloel, Maslin Masrom, Sani Susanto, Harto Tanujaya, Agustinus Purna Irawan, Ariawan Gunadi, Vikas Kumar, Jose Arturo Garza-Reyes, Tji Beng Jap, Frans Jusuf Daywin

**Affiliations:** 1 Industrial Engineering Department, Universitas Tarumanagara, Jakarta, Indonesia; 2 Industrial Engineering Department, Universitas Indonesia, Depok, Indonesia; 3 Razak Faculty of Technology and Informatics, Universiti Teknologi Malaysia Kuala Lumpur, Kuala Lumpur, Malaysia; 4 Industrial Engineering Department, Universitas Katolik Parahyangan, Bandung, Indonesia; 5 Mechanical Engineering Department, Universitas Tarumanagara, Jakarta, Indonesia; 6 Department of Law, Universitas Tarumanagara, Jakarta, Indonesia; 7 Faculty of Business, Law and Social Sciences, Birmingham City University, Birmingham, UK; 8 Centre for Supply Chain Improvement, University of Derby Business School, Derby, UK; 9 Faculty of Information Technology, Universitas Tarumanagara, Jakarta, Indonesia

**Keywords:** Blockchain, Simulation, Supply chain management, Risk mitigation, Pesticide industry, Energy science and technology, Engineering

## Abstract

This research was conducted on industrial agriculture in Indonesia. Risk analysis was carried out based on previous research. One source of risk was obtained, namely raw materials that did not meet specifications, which was then proposed to be mitigated by evaluating supplier performance. This activity involves a lot of data, requiring efficient and effective data storage and access. The level in the simulation layout includes analysing system needs, using problem diagrams, compiling activity diagrams, deciding subprocesses, and filtering information. The analysis is carried out by comparing the use of supply chains with Blockchain and without Blockchain, which is then obtained to determine whether there is an increase. A sequentially stored data scenario describes a situation when the transaction process is in progress and is stored sequentially according to the process that occurs. Storing data in groups explains a problem when a transaction has been completed and stored in groups with similar data, making it easier to track specific data. In this regard, a simulation will be carried out using a website, namely a blockchain demo. The design stage starts with identifying system requirements, creating use case diagrams, compiling activity diagrams, determining subprocesses, and selecting information. The simulation results obtained will be analysed to determine the feasibility of Blockchain as a means of supporting risk mitigation related to data using aspects, including security, trust, traceability, sustainability, and costs.

## Introduction

Blockchain technology uses decentralised systems, distributed computing, asymmetric encryption, timestamps, and consensus algorithms^1^. Therefore, the stored data cannot be changed or modified, so this technology can guarantee security and trust when making transactions^2^. With the growing interest in Blockchain, this technology has been widely implemented in many sectors and industries, including financial, business, industrial, voting, education, health^3^, supply chain management, healthcare information systems, e-government, voting systems, smart contracts, and digital signatures, has attracted the attention of early beginners^4^^,^^5–7^.

Blockchain technology enables organisations to develop new applications that can significantly improve the supply chain. Blockchain provides transaction transparency, data security, and seamless trust, reducing costs, creating more effective supply chains, and satisfying customers^8^. Blockchain enables supply chain partners to share data in a more trusted way, leading to improved collaboration. Blockchain will be a truly transformative technology for the supply chain industry^9^.

Recently, the rise of blockchain technology has overcome many problems with characteristics such as decentralisation, digital contracts, openness, encryption, and so on^10^ and can overcome supply chain problems with new traits. Moreover, in small and medium businesses, sharing information created via Blockchain has an extraordinary progress effect in improving the skills of various company resources and can increase productivity. At the current level, Blockchain has attracted much interest from experts and has been able to be applied in multiple fields. For example^11^, mentioned that Blockchain can trace and investigate the transparency and sustainability of supply chains in technological design. The agricultural supply chain system based on ^12^ obtained an overview of increasing business resource employment from the double chain framework of Blockchain^13^ investigated several operations management researchers regarding Blockchain’s influence. If the raw material does not meet the required criteria specifications, it cannot be used because it will reduce the quality of the finished material. What happens next is that the raw materials will be returned, and a refund will be requested. This action will affect the production schedule as it cannot meet the deadline set by the buyer^14^. Blockchain technology can empower supply chain resilience management^15^. The results or impacts of supply chain performance can be in all fields without exception^16^. Blockchain technology significantly impacts changing the manifestation of criteria in traditional supply chains^17^. For this reason, it is essential to investigate the methods used to measure new achievements in the supply chain using blockchain technology.

There is a lot of enthusiasm for applications in the current financial and digital currency markets, the most prominent of which is Bitcoin. Blockchain technology has excellent capabilities that can be applied to things far beyond cryptocurrencies^18,19^, and most people believe that blockchain technology can overcome many of these obstacles. Blockchain itself can be accessed by all customers with a specific network at any level of the supply chain to record everything to the ledger transparently in the supply chain sector in a complete manner^20^. Blockchain technology is becoming a big trend because it can provide significant and fundamental changes in systems capable of producing highly reliable, trust-based and integral infrastructure^21^.

The level in the simulation layout includes analysing system needs, using problem diagrams, compiling activity diagrams, deciding subprocesses, and filtering information. The feasibility of blockchain technology as a means of supporting risk mitigation, many factors will be used as a reference, including security, trust, traceability, sustainability, and costs. The analysis is carried out by comparing the use of supply chains with Blockchain and without Blockchain, which is then obtained to determine whether there is an increase. The purpose of this study is to identify the use of Blockchain for the pesticide supply chain, starting from the need to use blockchain technology, simulation design, data storage can work well through simulation results, and the feasibility of Blockchain as a means of supporting risk mitigation by analysing it on five different aspects.

## Literature review

### Block chain technology

Blockchain technology has great potential to solve three supply chain issues: traceability and transparency, counterfeiting, and efficiency play^22,23^. Blockchain technology introduces order, simplicity, trust, visibility, and automation to a chaotic environment. Blockchain dramatically streamlines paper-based processes while introducing greater security and visibility simultaneously. The technology digitally models real-world relationships across the global supply chain ecosystem. Blockchain holds details of each part and makes it accessible to each manufacturer in production. Blockchain enables firms to see across tiers in the supply chain, both upstream and downstream^24^. Blockchain is an alternative that can improve and speed up information sharing, replacing paper tracking and manual inspection systems that make supply chains vulnerable to inaccuracies^25,26^. This potential for information sharing can be seen as strengthening the overall ability to control the supply chain and its activities. Blockchain helps build and execute smart contracts, creating trading partner visibility and more efficient collaboration. Blockchain’s peer-to-peer transactions eliminate the need for intermediaries, reducing each transaction’s cost^27^. The technology enables a single point of contact for data, eliminates the central authority needed to validate transactions, allows decisions based on total supply chain information, and promotes collaboration with partners^28^. Additionally, Blockchain record-keeping procedures that keep track of transactional data in a secure, verifiable, and permanent produce a chain of records and ownership that is much less vulnerable to fraud and cybercrime and difficult to hack and alter. The technology establishes trust among partners by ensuring that every transaction is recorded and stored in multiple locations across the entire distributed network. Administrative functions will be drastically reduced or eliminated due to the increased visibility of transactions and the potential to avoid non-value-adding activities. In turn, it will improve supply chain efficiency and reduce system complexity. Blockchain also builds confidence in the journey of a product. Customers learn about what the product is made of, where it came from, and its impact on the environment. Producers and retailers benefit from better product tracking and empowering customers with s new information. Additionally, blockchain technology enables producers and retailers to get insight into what customers want and tailor their goods and services accordingly^29–33^. Figure 5.1 summarises real-world examples of Blockchain changing supply chain management^34^.

### Block chain technology in transaction ledger

Blockchain is a digital and distributed cash transaction ledger, recorded and reflected directly (real-time) on a computer network or node. There is no need for a central authority to authorise transactions; therefore, Blockchain is called a trustless peer-to-peer mechanism. Blockchain technology provides a more secure way for cash ledgers to store records and databases without centralised manual intervention. So, verifying transaction archiving in a blockchain-based system is much cheaper than in a centralised human verification system. The result is an operating model for transactions in the system based on system-based trust rather than counterparty-based trust^35^.

Blockchain technology can track every activity in the Blockchain, where every record of that activity is an activity that has been validated. Each block in the Blockchain contains data from all transactions in the system over a certain period of time and can create a digital signature that can be used to verify the validity of information related to the next and previous blocks. Transactions that have been carried out will be stored in blocks. If the transaction has been verified with a consensus of all or the majority of members in the network, then the transactions that have been stored in the block cannot be changed or deleted^36^.

### Agriculture business supply chain

#### Agrifood supply chain

In the agrifood supply chain (ASC), collaboration is often carried out by stakeholders at several different levels, from the farm to the hands of other customers, and the influencing variables have many similarities^37,38^. Throughout the supply chain (SC), many stakeholders try to increase the complexity of work, resulting in a lack of transparency and many obstacles related to this^39^. The need for transparent information in the supply chain is triggered by the food crisis and food safety^40^.

#### Pesticide supply chain

Pesticide supply chain actors in the Agriculture industry include raw material providers/suppliers, processing/production departments, distributors, retailers, and end consumers. The primary raw material providers/suppliers in active ingredients come from abroad, namely 80% from China and the remaining 20% from Japan, Belgium, Korea, Germany, and Malaysia. In contrast, auxiliary materials and packaging materials come from local domestic suppliers^41^. Related to these obstacles, Blockchain, with its characteristics, can provide benefits in the form of more transparent, secure, and trustworthy data between the parties involved^42^. With these benefits, Blockchain can be used as an auxiliary means of accessing and collecting data necessary for supplier performance evaluation, such as ease of data tracking, transparency, distributed data, and avoidance of data manipulation^43^. This benefit also applies to other risk mitigations related to data needs^44^.

### Block chain system

#### System requirements analysis

In designing a system, analysing needs needs to be done. Needs analysis is carried out to determine the specific needs of the system consisting of the outputs that must be produced, the inputs required to produce the output, the operations performed to produce the output, and the resources needed to make the system run and deliver output^45^.

#### Use case diagram

Use case diagrams are modelling to identify the behaviour of a system to be created. Use case diagrams illustrate the users in the system and what users can do to the system. Actors involved in use case diagrams have two characteristics, namely actors outside the system being developed and actors who interact with the developed system^46^.

#### Activity diagram

An activity diagram is a diagram that illustrates the natural dynamic nature of a system in the form of a flow model and control of activity to activity. The difference between activity diagrams and use case diagrams is that activity diagrams describe how a process runs. In contrast, use case diagrams illustrate how actors use the system to perform activities. In the activity diagram, a decision is used to describe behaviour under certain conditions. The advantage of using activity diagrams is that it makes it easy to understand the running scenario. Activity diagrams model workflow or business processes and operations internally. This diagram will describe processes in more detail than in the use case diagram^47^.

#### Subprocess determination

From the known processes, which subprocess will be supported by blockchain technology is selected. The selection is based on activity types full of cross-individual data transactions. The choice can also be found in the activities with the highest risk. Such increased risks need to be mitigated with a blockchain system. One form of risk is data misappropriation and manipulation^48^.

#### Information selection

Information selection is choosing which information from the activity will be supported by the blockchain function. This information must be carefully selected to prevent the system from managing too much data. Data needs to be made efficient without reducing the effectiveness of data transactions. The information chosen can be based on the system’s primary purpose of being applied to the Blockchain^49^.

#### Blockchain mechanism

In how Blockchain works, a block generally consists of 3 things: the data in the block, the hash of the block itself, and the previous block’s hash. A block must have a hash of its cryptography and the hash of the last^50^ to stay connected in a chain. If the hash changes, the block will be considered invalid in the Blockchain. A hash is a string returned from a mathematical function called a hash function. The hash function takes various inputs and converts them into fixed-length strings. Even if the change to the input string is tiny, the hash function will generate a new hash that may differ significantly from the actual result. Usually, the hash function used in blockchains is SHA-256. The longer the blockchain chain, the more complex the hash value to look up^51^.

### Supply chain management

Supply Chain Management (SCM) contains information, finance, and material flows that create close collaborative relationships between suppliers, manufacturers, vendors, and customers^52^. The main goal of the supply chain (SC) is to increase overall profits through cost reduction^53^. SC currently has several social-environmental issues and sustainability problems. The considerations for sustainability issues include several socio-economic-environmental dimensions in decision-making regarding climate change, which has become the main trigger for demand and can increase customer loyalty. The issue of sustainability in SC is driven by several reasons involving concentration on social, competitive and regulatory dimensions^54^. Consumers now have concerns about sustainability issues, which also puts pressure on manufacturers and suppliers to pay attention and work together on sustainability issues^55^. New technology also makes a significant contribution to the problem of implementing sustainability in the supply chain. One of the technologies that is currently widely applied is blockchain technology. Blockchain technology provides a new colour or significant revolution in supply chain sustainability^56^.

Increasing the sustainability of SC requires the application of BT because it has many valuable benefits. Investment limitations mean that the application of BT cannot develop rapidly. Privacy considerations remain hot among SC stakeholders because too much information sharing can unintentionally distort market structures^53^. Prevention of these complications must then be managed effectively, and data and privacy issues must be addressed. Worst of all, BT can become a tool for personal abuse of power and market control^52^. Another obstacle to using BT is its high energy consumption^57^. Repeated data input also causes BT energy consumption to be high because it has to increase computing power, thereby causing more carbon emissions^58^. So, there are many risks in implementing BT in SC sustainability. Studies must be conducted on this new technology’s internal and external challenges^59^. In SC sustainability, implementing BT involves many risks and challenges from various aspects and requires identifying and prioritising accuracy and essentials. The critical research to be carried out is to identify and provide the best solution for adopting BT in SC in implementing sustainable development factors. Limitations in the real world must be overcome to produce the best solution for implementing BT. One way to determine practical solutions is to use the Multi-Criteria Decision Making (MCDM) approach.

### Block chain in supply chain management

In a network that is used jointly and independently, decentralisation can be realised; Blockchain should be able to manage supply chain activities in a decentralised manner and with integrity, so this causes many to lose the need to collaborate with other third-party systems to support communication or several related parties. Others^60,61^. Another important thing in the blockchain system is the ability to show the location of an item very precisely at every point and record transactions that have been carried out thoroughly^62^. Context capability is another essential feature of Blockchain. This implies that if many parties are on the network, all data changes will be appropriately recorded^63^. This ensures companies have a high level of authentication regarding the origins of their products. This helps companies significantly reduce costs and time to detect violations and errors while managing data correctly and safely for the entire organisation^64^.

Several studies have been conducted in food supply chains, designed, developed and implemented in blockchain-based system solutions. However, there is a belief that the user’s subjective matters cannot be appropriately resolved precisely by understanding the opportunities and challenges in implementing the system. It is deemed necessary to conduct behavioural science studies in information systems (IS). According to the definition of Bariff and Ginzberg^65^, research conducted on information system behaviour will have a descriptive tendency, namely looking for systematic relationships between observations of user and personnel behaviour SI. Current trends in behaviour in information systems cannot be ignored by technology but are expected to be able to develop non-technical strategies that aim to change attitudes, management policies, and organisational behaviour related to technology^66^.

Although Blockchain has some significant capabilities that optimise productivity and reduce costs, it raises significant concerns that must be addressed. It poses barriers that executives must remove over time, and decision-makers can adapt to the widespread acceptance of this technology^18^ have concerns about a significant impact on technology adoption for managerial parties. Planning begins with creating a framework for appropriate blockchain adoption by professionals, who can determine the unique values associated with the organisation’s goals and culture. Another thing that could help is using alternative Design Science Research methods^67^ to design an artefact to address requirements and determine the most critical priorities regarding trust at each point in the agrifood supply chain. This framework can make implementing a blockchain-based information system for food tracking easier in real-time.

## Methods

**Figure 1:**
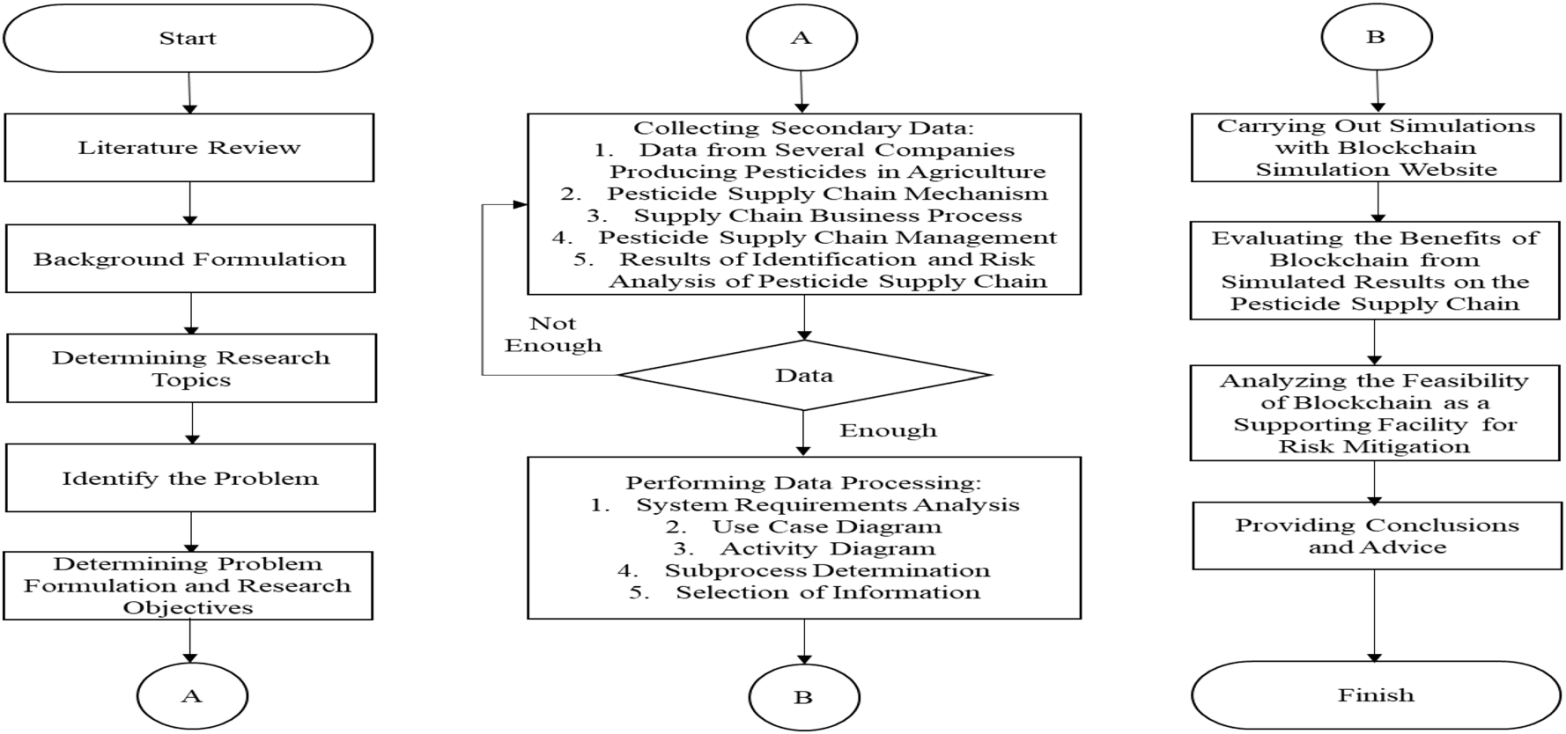
Method flowchart.

The research method was carried out by holding discussions with informants with expertise in this study’s data and data assessment field. Then, secondary data in the form of historical data is obtained employing literature studies originating from the company’s official website, research, and publications that have been carried out at the company. The data collected includes general company data, supply chain mechanisms, business processes, and management. The flowchart of the research method can be seen in Fig. 1.

The research method is described as follows: 1. Literature study of scientific journals and proceedings related to the researcher’s topic of interest. With this study, researchers can enrich themselves in knowing developments, problems and research gaps that can be researched; 2. The background formulation is carried out by the findings obtained by researchers from various sources that have been obtained, then compiled so that potential problems that need to be overcome can be found; 3. From the background that has been prepared, ideas are obtained regarding potential problems to be used as research topics that will be studied further; 4. Based on the background, problems can be found so that potential problems are identified further and clearly, and specific problem identification is obtained; 5. Then, the problem formulation and research objectives are determined so the research becomes more directed and focused; 6. Next, secondary data was searched and collected according to data relevant to the researcher’s research, namely general company data, pesticide supply chain mechanisms, pesticide supply chain business processes, pesticide supply chain management, and the results of chain risk identification and analysis. pesticide supply; 7. If the required data is sufficient, data processing can be carried out, including analysing the system’s needs under study, creating use case diagrams, compiling activity diagrams for the system, determining the subprocesses that will be simulated, and selecting information that will become simulation data; 8. Carrying out blockchain simulations according to data processed using third-party tools in the form of a blockchain simulation website, namely Demo Blockchain (https://demoblockchain.org/).

Apart from that, discussions were held with four sources to increase the research’s objectivity. All resource persons are academic experts with experience and expertise related to the problem being studied. All the steps in the research methodology should be addressed carefully to guarantee objectivity and reach the research aims. The okay (✓) criteria get a minimum of 50% scores from the expertise (a minimum of two people agree with the requirements of the subprocess).

This research used the explanatory case study method^68^ mixed with qualitative and quantitative evidence to illustrate specific topics within an evolution in blockchain technology. The role of theory in this research is essential to construct a preliminary theory and requires theoretical propositions. The rationale for a single-case study represents unique circumstances. Analysis Technique with Logic models in Repeated cause-effect relationship with individual level or organizational-level logic model. The structure reporting with comparative analysis repeats the same case study two or more times, comparing alternative descriptions or explanations. This case study’s identity as real and anonymous with full disclosure is the most desirable option, helping readers link in previous research and ease of review.

## Data collection

Data was collected in 2020 by Agusti and Mulyati^69^. The agriculture industry is the companies that produce pesticides in Indonesia. Based on previous research on the pesticide supply chain in this industry, a risk analysis was carried out, which then obtained the results of one of the risk agents, namely raw materials not meeting specifications. The source of this risk is included in the high-risk classification, which is a priority to be addressed. For these risk mitigation alternatives, it is proposed to evaluate supplier performance, which will involve a lot of data, so efficient and effective data storage and access are needed. The supply chain of the Agriculture industry still processes data independently by each actor and shares data that is considered necessary when coordinating in traditional ways, so this has the potential for data manipulation due to low transparency. Data can be stolen due to weak security and requires a short time in the data exchange process.

### Material requirement planning in the pesticide industry

**Figure 2:**
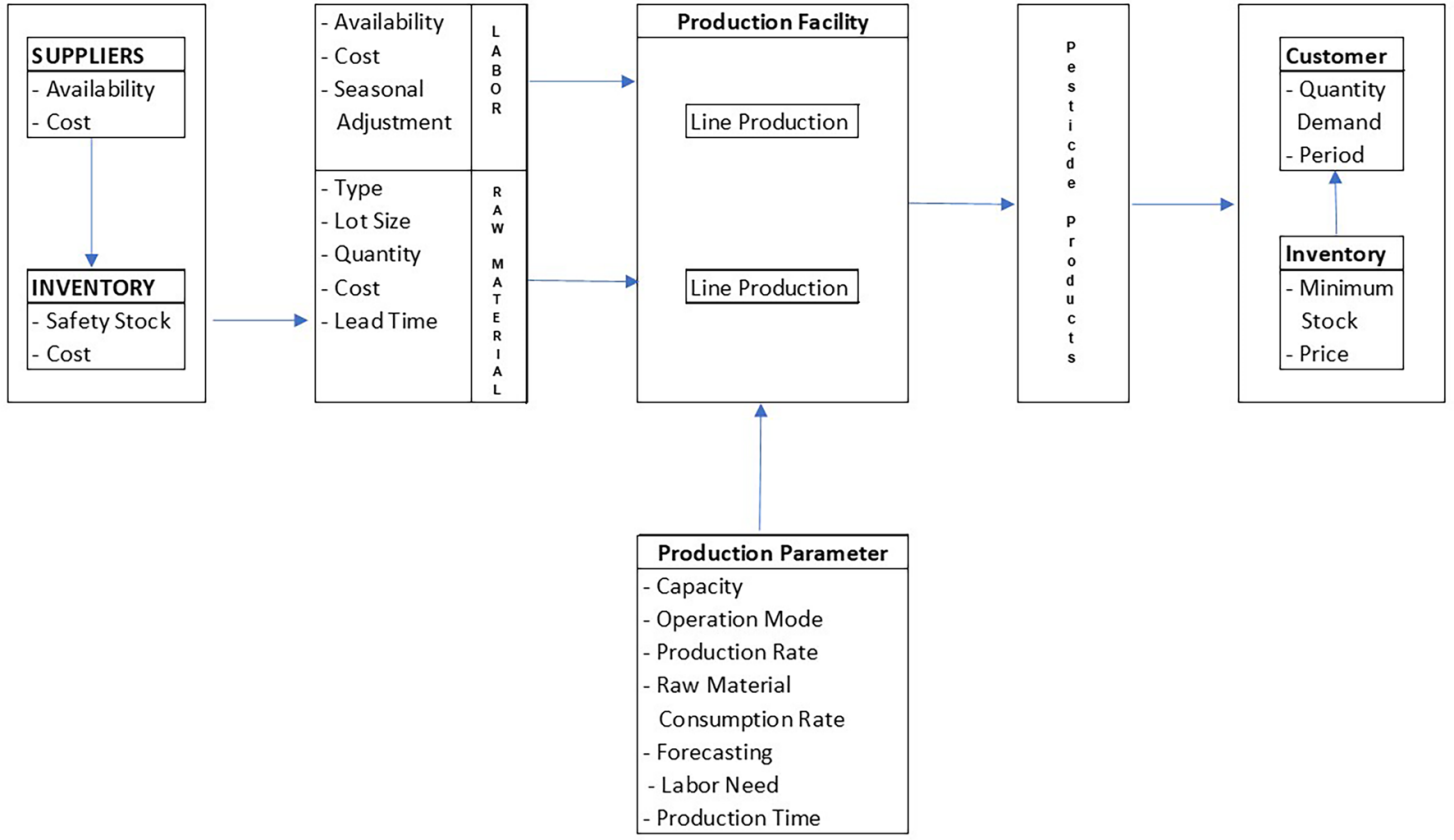
Pesticide production system.

The company produces pesticides, fungicides, insecticides, and herbicides. The demand level is uncertain and not fixed per day, and the company is required to plan the production process sequences precisely to meet consumer needs. The company experienced problems such as high shipping and ordering costs, and raw material inventory was far less than inventory capacity^70^. The company must maintain raw materials availability, such as a 1 Liter insecticide packaging bottle, to expedite the production process. It was scheduling a 1L insecticide packaging bottle as its superior product needs to be done to determine the inventory amount in a certain period. The inventory can meet demand, knowing the safe amount of inventory to meet production needs optimally, and minimizing ordering costs by maximizing ordering capacity. The pesticide production system is described in Fig. 2. The pesticide production system chart created is refined from the chart originally created by Elimam^71^. The additions elements provided include safety stock, lead time, lot size, forecasting, production time.

### Pesticide supply chain mechanism

**Figure 3:**
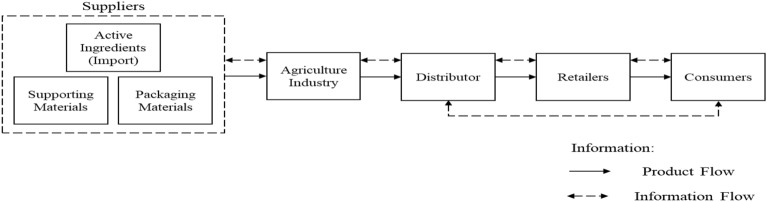
Structure of the pesticide supply chain network.

The agriculture industry has suppliers that provide the main ingredients from abroad, namely 80% from China and 20% from Japan, Belgium, Korea, Germany, and Malaysia. As for the suppliers of supporting materials and packaging materials, they come from local/domestic. The Agriculture Industry has market coverage focusing on generic companies and retailers. The structure of the pesticide supply chain network in the Agriculture Industry can be seen in Fig. 3.

### Pesticide supply chain management

**Figure 4:**
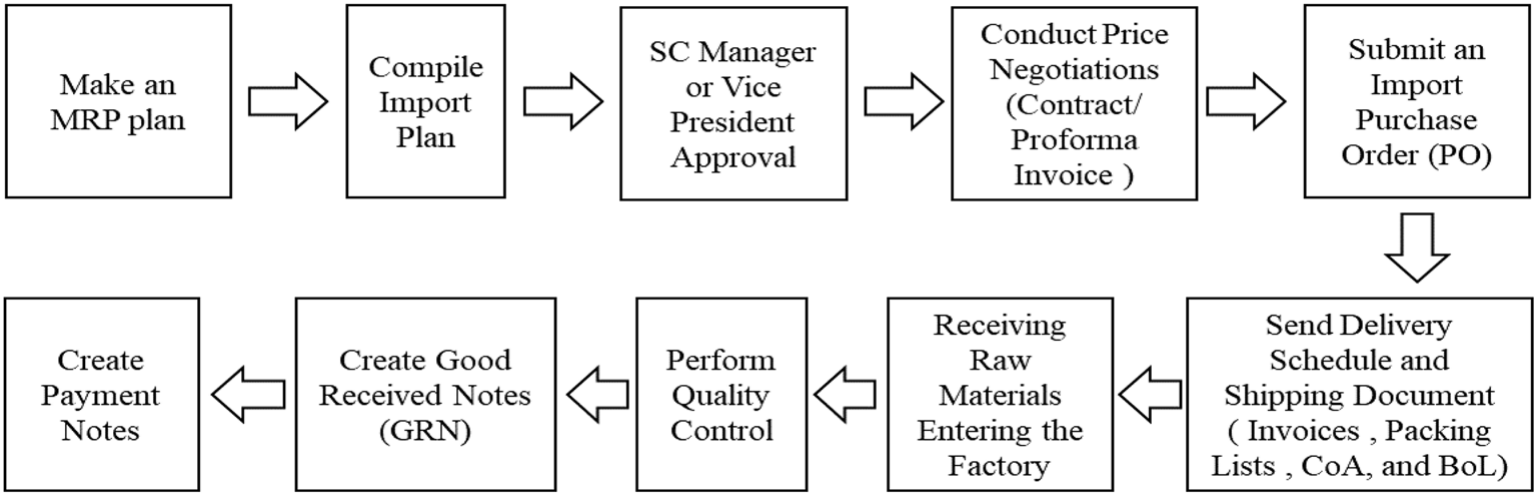
Transaction flow of raw material procurement process.

The pesticide supply chain transaction system is divided into the transaction system in the procurement of raw materials and the transaction system in the product sales process. A transaction system can be used to procure raw materials to evaluate supplier performance. The flow of transactions in the raw material procurement process can be seen in Fig. 4.

The following is an explanatory description of the transaction flow in the raw material procurement process.

- MRP and forecasting planning is carried out at the beginning of the raw material procurement process, and the planning is related to production planning and inventory control.
- Import Plan is an import plan related to the company’s strategy and procedures for importing goods, including selecting suppliers.
- Approved by the SC Manager or Vice President before implementing the plan. The SC Manager or Vice President must know it first and approve it.
- Price Negotiation: Carrying out price negotiations means making a contractual agreement for a new supplier entering into a contract for the first time. Meanwhile, suppliers who have become subscribers use proforma invoices.
- Import Purchase Order (PO): after an agreement has been reached with the supplier, a PO is made to order the required raw materials from overseas suppliers.
- Delivery Schedule and Shipping Document: when the supplier has received the PO and the goods are ready to be sent, the delivery schedule will be known along with the documents, including invoice, packing list, CoA, and BoL. Invoices contain transaction details between sellers and buyers. The packing list includes detailed information about the goods sent. CoA (Certificate of Analysis) is a document that provides laboratory test results related to product specifications, such as content, composition and quality. BoL (Bill of Lading) is proof of a transportation contract, goods receipt, and ownership document in sea shipping.
- Raw materials entering the factory, receipt of raw materials by the factory after the goods arrive at their destination.
- Quality Control: raw materials will be checked first through quality control by QC before being stored in the warehouse.
- GRN (Good Received Note) by the warehouse department, it is necessary to ensure that the goods received are in accordance with what was ordered. GRN functions as official proof of goods receipt and validating suppliers’ invoices.
- Payment Note is a document that details the payment transactions carried out. Has a function as proof that the buyer has made payment.

**Figure 5:**
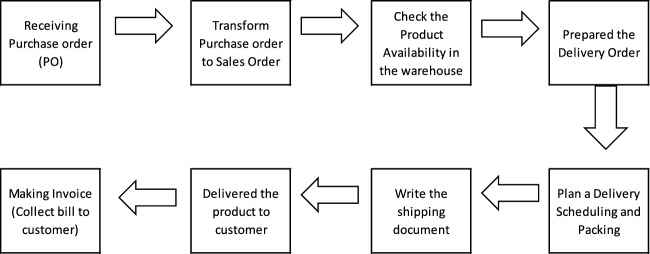
Transaction flow in the pesticide product sales process.

Meanwhile, the transaction flow in the sales process for pesticide products can be seen in Fig. 5:

The following is an explanatory description to understand better the transaction flow in selling pesticide products.

- Logistic Care receives Purchase Order (PO). Incoming orders from buyers are accepted as a PO document. This document shows the agreed type, quantity and price of goods.
- Sales Order (SO): After the company receives the PO, the document will be converted into an SO, indicating that the company has approved the buyer’s order to sell the product according to the PO received.
- Check product availability in the warehouse. After the company agrees to fulfil the PO from the buyer, a stock check of the product ordered will be carried out.
- Delivery Order (DO): if the quantity of the product ordered can be met, the company will issue a DO document to the warehouse to release the goods to the buyer.
- Scheduling Delivery and packing: the goods to be sent need to be scheduled so that Delivery can be carried out regularly. Apart from scheduling, it is also necessary to pack goods to protect them from damage during shipping and make transportation easier.
- Travel document: When the goods are ready to be sent, they will be accompanied by a travel document. It functions as proof of Delivery and contains information, such as details of the goods, sender and recipient.
- For the Delivery of products to consumers, products are sent according to a schedule and require a specific time to REACH their destination, depending on the distance between the buyer and the seller.
- Invoice (billing to consumers): after the goods are received by the buyer/consumer, payment will be made for the goods ordered in the form of an invoice.

## Results and discussion

### Simulation design

Simulation design helps obtain data for use in simulations. The following is a simulation design consisting of stages: analysing system requirements, creating use case diagrams, compiling activity diagrams, determining subprocesses, and selecting information.

1. System requirements analysis

In carrying out a system requirements analysis, it is necessary to know in advance a system analysis of the needs for using blockchain technology. The study results can be seen in Table 1.

**Table 1:** Results of needs analysis for using blockchain technology.

Questions	Answers
Is there data or information that needs to be stored?	Yes, in the flow of pesticide supply chain transactions, some data need to be recorded and stored correctly, especially external data, because data exchange occurs between supply chain actors, such as purchase orders, invoices, payment notes, etc
Is there more than one writer that stores data on the system?	Yes, the data writers have data because the records are carried out by actors who play a role in the pesticide supply chain process, including suppliers, pesticide companies, distributors, and retailers
Are there trusted and active third parties?	No recording is carried out independently by each actor in the pesticide supply chain, and data is only shared between actors when necessary
Is it necessary to know the entire role of the author who stores data on the system?	Yes, because all the authors are actors involved in the pesticide supply chain. Therefore, it is necessary to know the identity of each actor who does the writing. The identity in question is the actor’s role in the pesticide supply chain, for example, supplier A, distributor B, retailer C, and so on
Can all authors on the system be trusted?	No, related to this, there is a potential that there are still writers who commit violations and are not acting honestly, such as manipulating data

**Figure 6:**
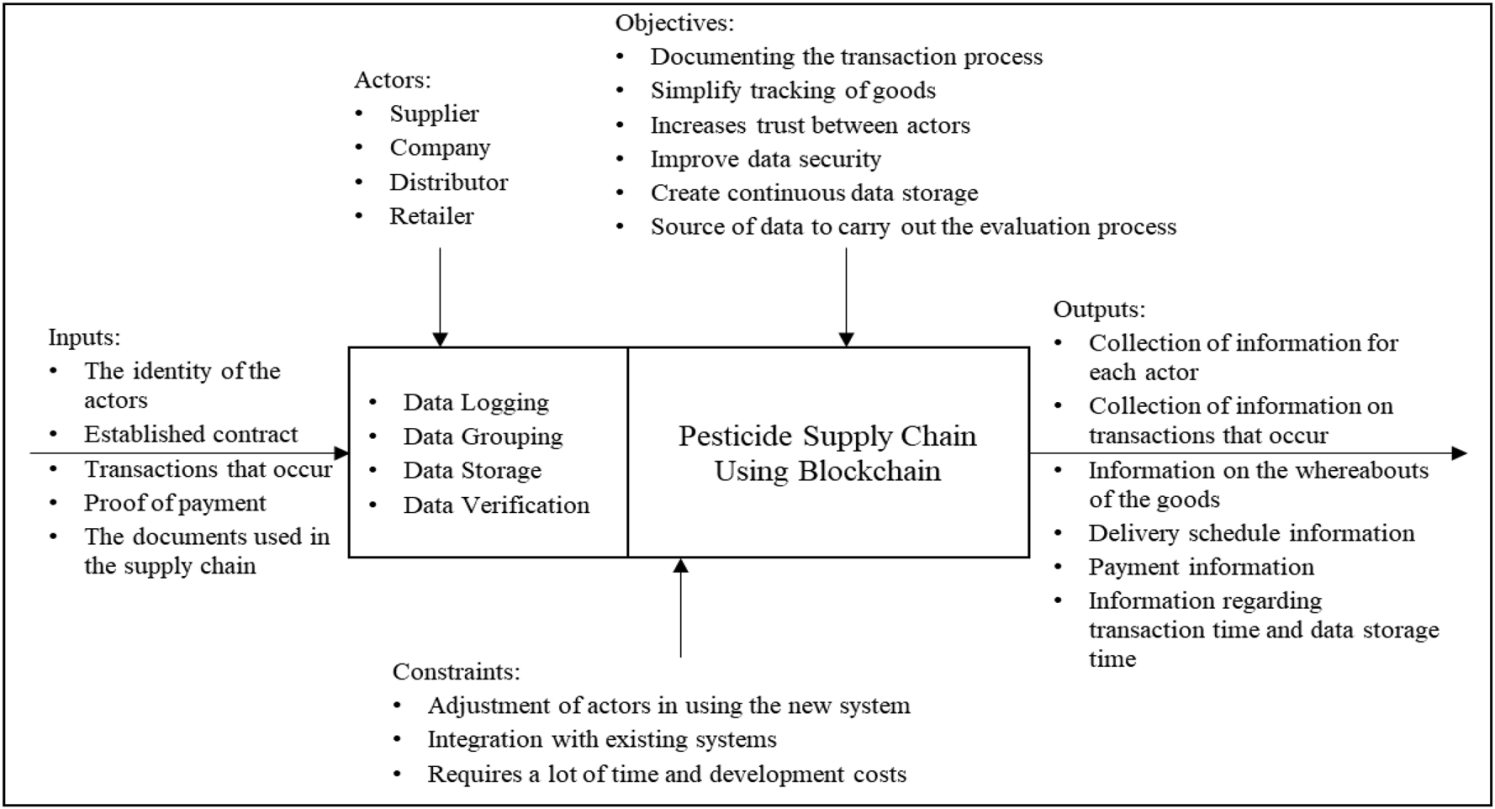
System requirements analysis.

Based on the results of Table 1, the blockchain technology suitable for recording data on the pesticide supply chain is a blockchain with private permissions. After that, system requirements were analysed, as seen in Figure 6.

2. Use case diagrams

**Figure 7:**
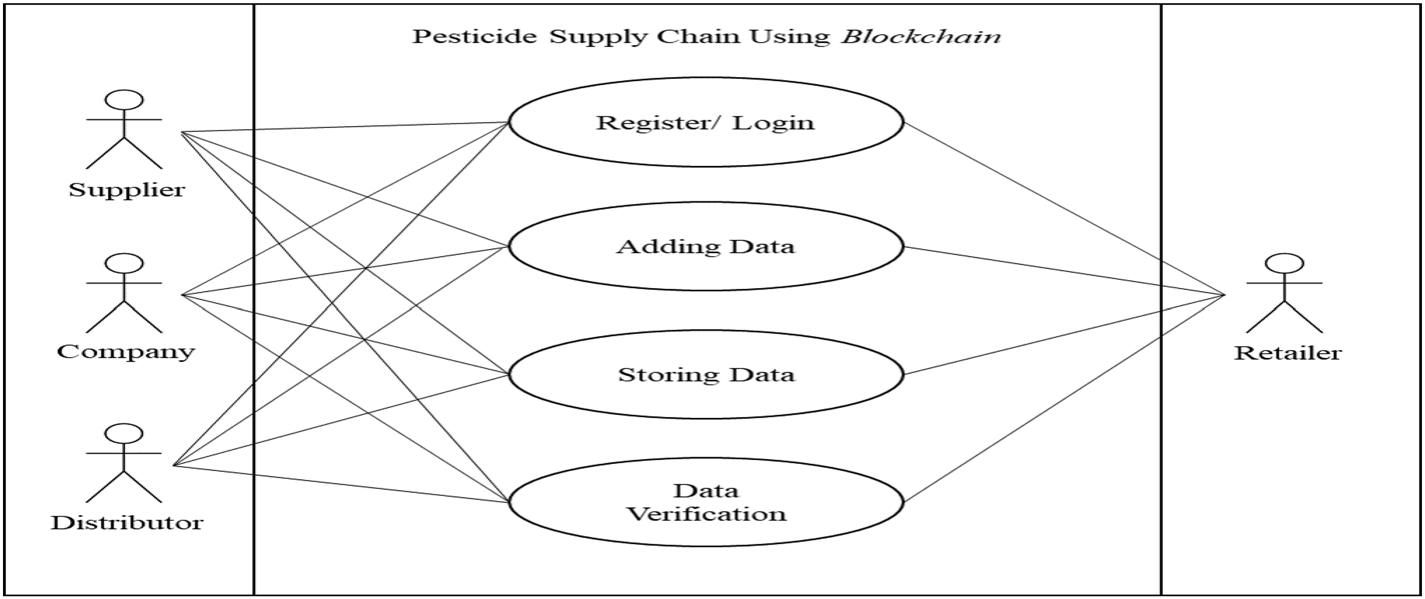
Use case pesticide supply chain diagram with blockchain.

Four actors are involved in the system: suppliers, companies, distributors, and retailers. On the left side of the diagram is a group of sellers, while on the right is a group of consumers. The use case diagram of the pesticide supply chain with Blockchain can be seen in Figure 7.

3. Activity diagrams

**Figure 8:**
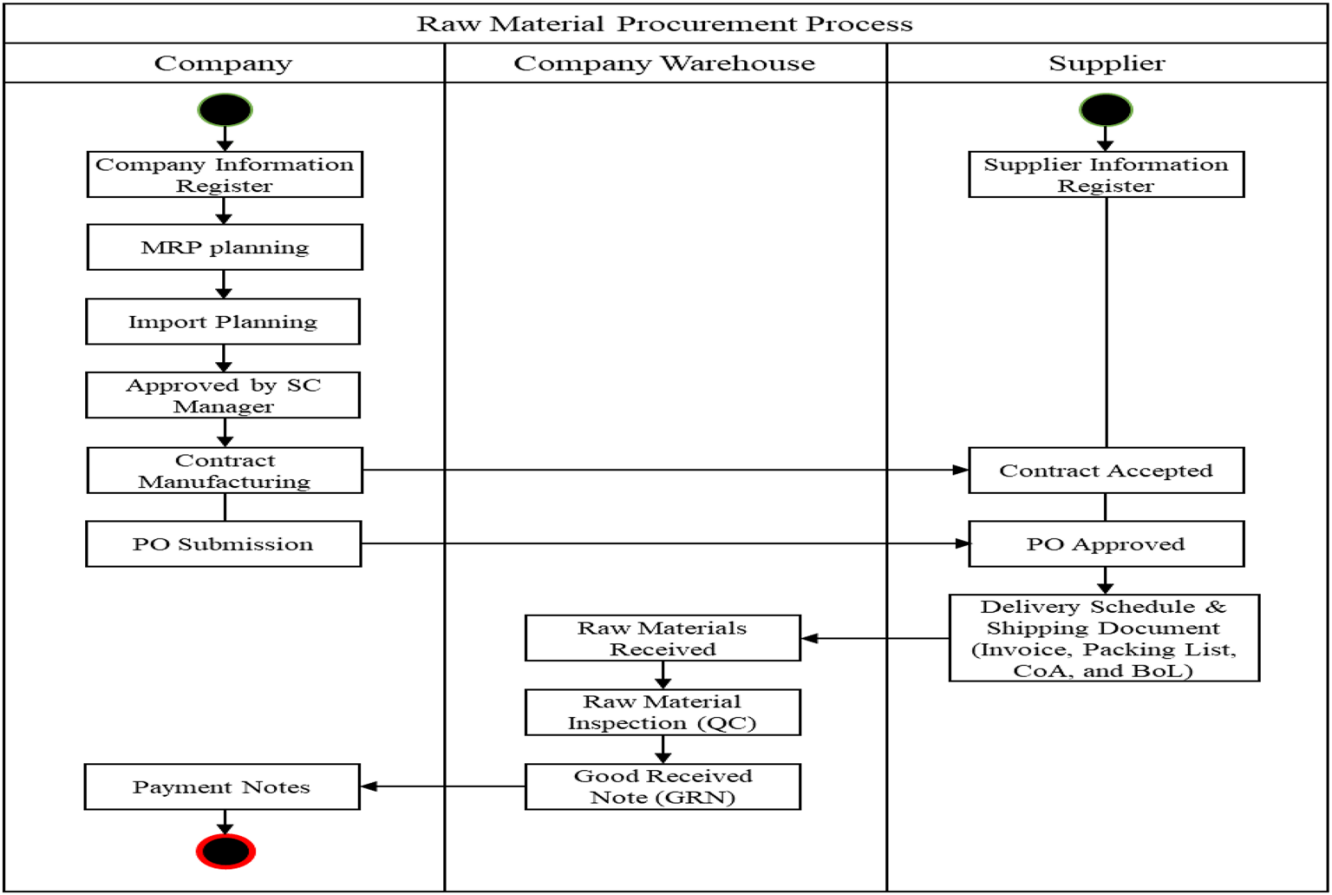
The activity of pesticide supply chain diagram with blockchain.

In procuring raw materials, only two actors are involved (company and supplier). Still, to clarify the activity, a company warehouse is added to receive and inspect the raw materials obtained. The company includes the PPIC, purchasing, and finance sections. The activity diagram of the pesticide supply chain with Blockchain in the raw material procurement process can be seen in Figure 8.

4. Subprocess determination

Determining sub-processes begins by looking at all the sub-processes that occur, defining sub-processes that contain a large amount of information and have significant impacts, and exchanging data with other actors in the pesticide supply chain. The subprocess selected for the simulation was determined by four sources with their respective roles and expertise through discussion. The okay (✓) criteria get a minimum of 50% scores from the expertise (a minimum of two people agree with the requirements of the subprocess). The sub-processes used in the simulation are price negotiation (contract/proforma invoice), PO (purchase order), delivery schedule & shipping document, and payment note. The following results from determining the subprocess which can be seen in Table 2.

5. Information Selection

**Table 2:** Subprocess determination results.

Subprocess	Data exchange with other actors	Information content	
		Lots	Simply impactful
Company information register^72^	✗	✗	✓
Supplier information register^95^	✗	✗	✓
MRP and forecasting planning^96^	✗	✓	✗
Import planning^97^	✗	✓	✗
Service control manager approval^15^	✗	✗	✗
Price negotiation (contract/proforma invoice)^98^	✓	✓	✓
PO (purchase order)^99^	✓	✓	✓
Delivery schedule and shipping document (invoice, packing list, CoA, and BoL)^100^	✓	✓	✓
Receiving raw materials^101^	✗	✗	✗
Raw material inspection (QC)^102^	✗	✓	✗
Good received note (GRN)^103^	✗	✓	✓
Payment notes^104^	✓	✓	✓

The selected information will focus on parts that are considered essential and can represent documents contained in specific sub-processes. The following is the information used.

- Contract:
  Name and position of party 1 (one) and party 2 (two), then articles in the document such as general provisions, types of goods, scope, rights and obligations, and implementation.
- Proforma Invoice:
  Document number, seller data, buyer data, shipping method, payment method, type of goods, quantity, and total price.
- Purchase Orders (POs):
  PO number, document date, PO status, supplier data, buyer data, number of items, item code, total price, and time of payment.
- Invoices:
  Invoice number, billing and shipping address, delivery date, shipping method, item description, and total price.
- Payment Notes:
  Date, billing number, billing date, billed total, payment method, payment details, and payment amount.

### Simulation results

The simulation uses third-party tools such as website-based Blockchain Demo software (https://demoblockchain.org/). The simulation is carried out in two scenarios: data stored sequentially according to the supply chain process and in groups according to each category. A sequentially stored data scenario describes a situation when the transaction process is in progress and is stored sequentially according to the process that occurs. Meanwhile, storing data in groups explains a problem when a transaction has been completed and stored in groups with similar data, making it easier to track specific data.

**Figure 9:**
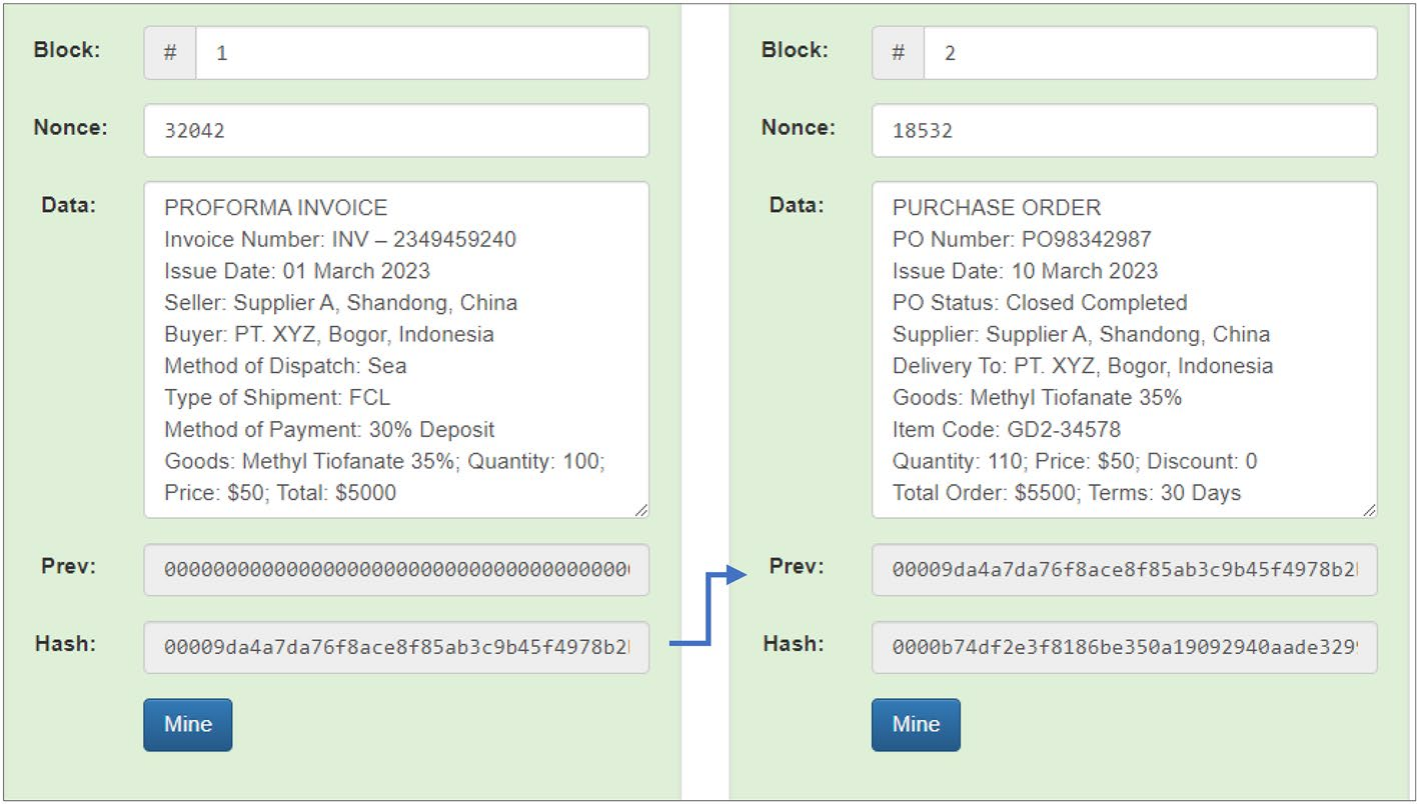
Sequential simulation results in Block 1 and Block 2.

In sequential scenarios, 4 (four) different documents are stored connected to each other in 4 separate blocks: proforma invoices in block 1, purchase orders in block 2, invoices in block 3, and the payment note in block 4. Block 1 has a previous hash value of “000000000000000000000000000000000000” because it is the initial block and managed to produce a hash value of “00009da4a7da76f8ace8f85ab3c9b45f4978b2”. Then the hash generated in block 1 becomes the previous hash in block 2 (two), as indicated by the arrow, and has succeeded in producing a hash value of “0000b74df2e3f8186be350a19092940aade329”. Sequential simulation results of blocks 1 (one) and 2 (two) can be seen in Fig. 9.

**Figure 10:**
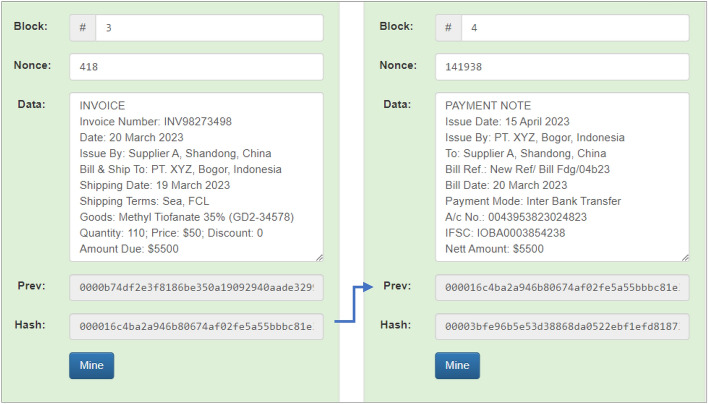
Sequential simulation results in Block 3 and Block 4.

Likewise, block 3 (three) and block 4 (four) also make the hash of the previous block the previous hash of that block. Block 3 succeeded in producing a hash value of “000016c4ba2a946b80674af02fe5a55bbbc81e”. Block 4 succeeded in creating a hash value of “00003bfe96b5e53d38868da0522ebf1efd8187”. Sequential simulation results of block 3 (three) and block 4 (Four) can be seen in Fig. 10.

**Figure 11:**
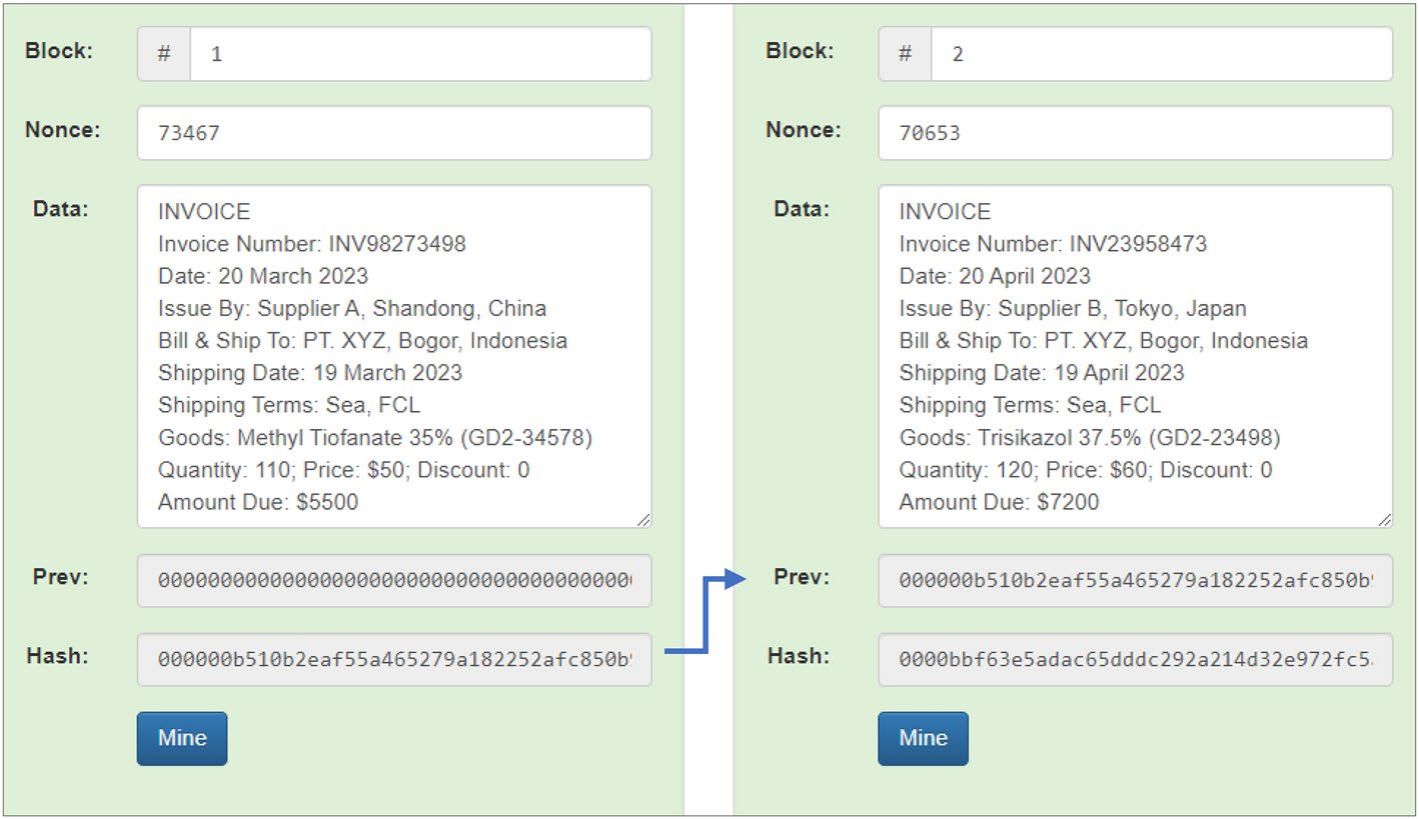
Invoice simulation results in Block 1 and Block 2.

Furthermore, invoice documents will be used for group scenarios to represent other documents. Four supplier invoice documents are stored and connected to each other in 4 blocks, namely blocks 1, 2, 3, and 4. Block 1 has a previous hash value of “0000000000000000000000000000000000000” because it is the initial block and succeeded in producing a hash value of “000000b510b2eaf55a465279a182252afc850b”. Then the hash generated in block 1 becomes the previous hash in block 2 (two), as shown by the arrow, and has succeeded in producing a hash value of “0000bbf63e5adac65dddc292a214d32e972fc5”. The simulation results for blocks 1 (one) and 2 (two) can be seen in Fig. 11.

**Figure 12:**
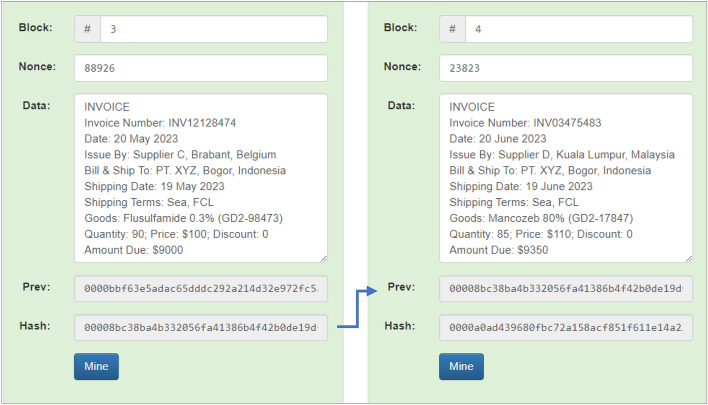
Invoice simulation results in Block 3 and Block 4.

Likewise, block 3 (three) and block 4 (four) also make the hash of the previous block the previous hash of that block. Block 3 managed to produce a hash value of “00008bc38ba4b332056fa41386b4f42b0de19d”. Block 4 succeeded in creating a hash value of “0000a0ad439680fbc72a158acf851f611e14a2”. The simulation results for blocks 3 (three) and 4 (four) can be seen in Fig. 12.

### Blockchain feasibility analysis as a means of supporting risk mitigation

The simulations that have been carried out show that the pesticide supply chain uses Blockchain for data storing running well. In both processes, procuring raw materials and selling products, the data contained therein can be reserved by generating the previous hash and hash of each block in the Blockchain. Supplier performance evaluation can only use the raw material procurement process related to contract documents, proforma invoices, purchase orders, invoices from suppliers, and payment notes to support risk mitigation. As a result of the feasibility of blockchain technology as a means of supporting general risk mitigation related to data, several aspects will be used as a reference to see it from various angles. The aspects used include security, trust, tracking, sustainability and cost^72^. The following is a description of each aspect used.

#### Security

**Figure 13:**
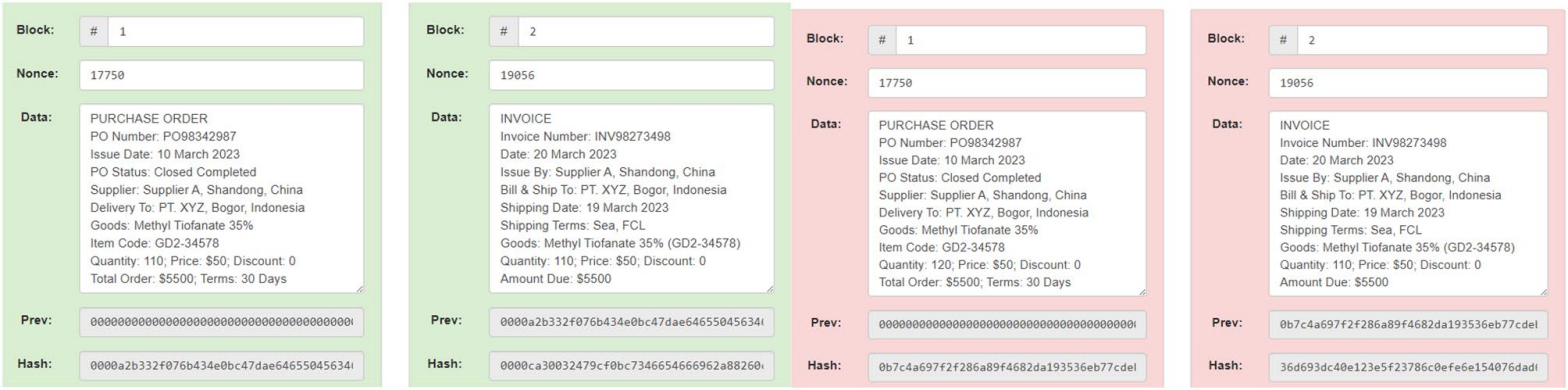
The red colour shows the data manipulation in data submission.

On the security side, pesticide supply chain data stored using Blockchain can be accessed by all supply chain actors, so it can also be called decentralised or distributed. This security side means that each actor has the same rights and authority in interacting with the system, and no one has higher or lower power than others. So, all actors have equal and fair power and can prevent attacks on the system because it cannot be attacked through just one point. Apart from that, the stored data is also distributed to all supply chain actors so that each actor has an exact copy of the data as each other. This stored data can prevent data corruption, resulting in data loss. Another thing that can be gained from the security side is the hash generated by each block. With this hash, any change to the data, no matter how small, will produce an invalid hash. In the end, data manipulation will be challenging to do. As a result of how this system works, data before and after the change occurs are shown in Fig. 13^73–76^. The change is a tiny entity, only differing by one number in quantity, and other data has no change. However, it can be easily seen that the resulting hash is immediately very different and is marked by the block’s colour changing to red where it was originally green. Even though data changes are only made to block 1, all subsequent blocks to the newest block will produce an invalid hash. So, when changes are made to the data in any block, no matter how small, the changes will result in a consecutive invalid hash up to the latest block. So, it will be effortless to see if data has been changed/manipulated.

#### Trust

**Figure 14:**
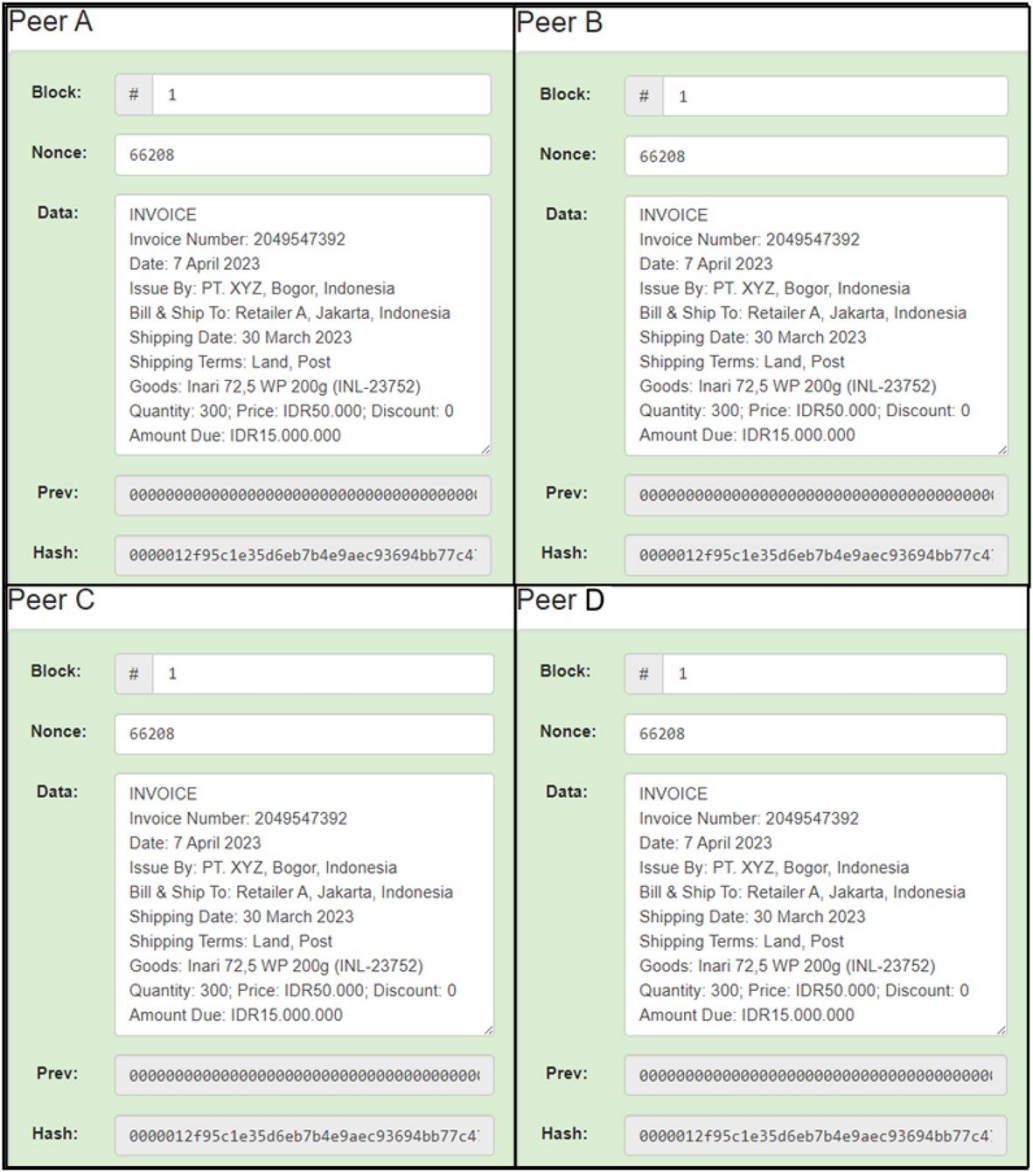
Data storage transparency between supply chain actors.

Blockchain allows all supply chain actors to have the same power level so that trust becomes dependent on the system, not on specific people with a higher access level. Of course, suppose the actor’s trust is based on the system. In that case, there is no need to worry about data manipulation because the system mechanism is resistant to data manipulation. In addition, the level of transparency in the pesticide supply chain is high because all supply chain actors have a copy of the same data and update each other^58,77–81^. The transparency of data storage in the pesticide supply chain can be seen in Fig. 14.

From Fig. 13, the four actors are depicted as Peer A, Peer B, Peer C, and Peer D, and each actor has an exact copy of the data. This data copy is also indicated by the same hash value for each actor, namely “00001c33ec8c1f4171a00d7a6b4a24cb4da780”. So, the stored data will be transparent between supply chain actors so that the level of trust can increase.

#### Tracking

Regarding data tracking, all data will be recorded sequentially and in categories. With a high level of transparency where data is distributed so that every actor in the pesticide supply chain has an identical copy of the data, inevitably, the stored data does not contain variations. This transparency can make data tracking easier when obtaining complete data because there is no need to re-coordinate between actors. In addition, data that has been stored is challenging to change, so all data has been well organised and stored, and its authenticity is guaranteed, so there is no need to verify data between actors. Of course, coordinating and verifying actors requires a lot of time and is quite a complicated process. That way, data tracking will be done in a shorter time. So, the data tracking carried out can be ensured to be accurate data tracking, and the time required is shorter. This makes data tracking more efficient and effective^82–86^.

#### Sustainability

In terms of sustainability, the stored data will last for an extended period of time. The data has been stored and well organised, and the authenticity of the data has been confirmed without verification because data changes are challenging to make, making it resistant to data manipulation. Then, trust in the system mechanism creates a high level of trust between pesticide supply chain actors, and there is no need to worry about unfair treatment or fraud in the system because it is not based on trust in someone. Then, data tracking that is more efficient in terms of time and effective in terms of data accuracy can contribute to improving the sustainability of the pesticide supply chain system. In addition, all actors have identical copies of the data, making the data resistant to corruption that, makes the data disappear and not be recovered. So, by using Blockchain, data can be stored continuously more efficiently and effectively without worrying about fraud between actors, data being lost, and data being manipulated^42,56,87–89^.

#### Cost

Blockchain technology is relatively new, requiring much money for design and development. Apart from that, costs are also needed to integrate this into the existing system in the pesticide supply chain. Don’t forget that when applying this technology to the system, there needs to be an adaptation from all actors in the pesticide supply chain to run the system correctly and obtain optimal results. In this adaptation, training is required for employees, so it requires training costs and a short amount of time to be able to fully adapt to this new system^82,90–94^.

Blockchain technology as a means of supporting risk mitigation in general related to data in the pesticide supply chain, several aspects will be used as a reference so that it can be seen from various sides. The aspects used include security, trust, traceability, sustainability, and cost. The analysis is carried out by comparing the use of Blockchain and without Blockchain, which is then obtained to determine whether there is an increase. The following is a comparison that can be seen in Table 3.

**Table 3:** Comparison of the use of blockchain in the pesticide supply chain.

Criteria	Without blockchain	With blockchain	Enhancement	
			Yes	No
Security	Processed by authorised persons, vulnerable to data manipulation	In a decentralised system, all actors have equal authority, which makes it challenging to manipulate	✓	
	Centralisation is susceptible to a single point of failure, resulting in lost data due to damage stored at only one point	Distributed, each actor has an identical copy of data, and it is easy to view data changes		
		The hash mechanism makes all data connected and sensitive to the slightest change, guaranteeing the authenticity and integrity of the data		
Trust	Based on humans, there is the potential for unfair things to happen	Based on a system with a reliable mechanism can avoid unfair things	✓	
	It requires high integrity from every actor to ensure every process runs without fraud	High transparency, easy to detect when changes occur and increase trust between actors		
Tracking	Collecting data requires coordination and verification, so it takes a long time	Distributed, all actors have an identical copy of data, avoiding variations in the data, so there is no need for coordination, which results in tracking data more efficiently in terms of time	✓	
	Each actor stores data, variations may occur, and specific data stored by only one actor may be lost	With data that is difficult to change, all data is guaranteed to be authentic and intact, with no need for verification, so that tracking remains accurate in a shorter time		
Sustainability	Data is stored separately by each actor, so there is potential for manipulation or data loss in the long term	Data is stored, decentralised, and distributed, resistant to long-term manipulation and data loss	✓	
	Trust is based on humans; integrity changes can occur in different generations	Trust is based on a system, using a reliable mechanism and always in a neutral position		
	Data tracking is less efficient in terms of time because it requires coordination and verification between actors	Data tracking is more efficient regarding time and accuracy because it does not require coordination and verification between actors		
Cost	The system is mature and operates in the pesticide supply chain for a certain period of time, so it only requires maintenance costs	The relatively new technology requires significant design and development costs to be integrated with existing systems		✓
	All actors are familiar with the existing system and conduct training only for new employees	All pesticide supply chain actors need to adapt, which requires a lot of time and employee training costs money		

Table 3 shows that Blockchain is appropriate to support risk mitigation related to data in the pesticide supply chain. Through the five aspects above, there are improvements in four aspects, namely security, trust, tracking, and sustainability. However, from a cost perspective, some challenges must be overcome through a large allocation of costs and time. However, considering that the agriculture industry is mature and has become a holding company, the challenges related to costs have a high probability of being overcome. This cost is also supported by the need for a pesticide supply chain, which is quite complex because it has a relatively large number of suppliers, most of which come from abroad. Likewise, the number of retailers is widely spread in various cities. The use of Blockchain as a means of supporting risk mitigation in the pesticide supply chain will provide good benefits.

## Conclusion

Blockchain has shown its potential for transforming traditional industry with its key characteristics: decentralization, persistency, anonymity and auditability. Through the simulation design that has been designed, it is obtained that the pesticide supply chain requires the use of blockchain technology, and the use of Blockchain in the pesticide supply chain can be applied following system requirements analysis, use case diagrams, activity diagrams, subprocess determination, and information selection that has been carried out. The simulation results show that data can be stored, organised, and connected correctly, as evidenced by the hash successfully generated by each block, and the hash then becomes the previous hash contained in the next block. The feasibility of blockchain technology to be used as a means of supporting risk mitigation related to data in the pesticide supply chain is declared feasible according to the results of a comparative analysis between supply chains without Blockchain and with Blockchain, which is carried out based on five aspects, namely security, trust, traceability, sustainability, and cost. Even though the cost aspect is a challenge that needs to be faced, the agriculture industry, with a mature company and a holding company, certainly has a high possibility of overcoming this. It is different if the company is still new or in bad condition; further consideration is needed. So, blockchain technology can be used to support data-related risk mitigation. Still, it is necessary to pay attention to the needs of the company’s supply chain and its readiness because of its condition.

## Future research

Many open issues still need to be researched and analysed to create more workable and practical industrial applications, healthcare, Real estate, consumer and civilian applications, government and military operations, and highly fast-moving products that can fully benefit from Blockchain technology and achieve the intended goals. About the legal action, different industries have different standards and security policies. The high operation cost of Blockchain is still the most challenging problem for blockchain applications. Besides high costs, some open issues include security, privacy, scalability, energy and mining issues, integration with other systems, and, more specifically, regulatory issues. It still needs quantitative analysis to review the performance and security of public, private and high-complexity companies. Most projects are in an early development phase, and research is still ongoing on key improvement areas that would allow desired scalability, decentralisation and security. Additional research initiatives, trials, projects and collaborations will show if the technology can reach its full potential, prove its commercial viability and finally be adopted in the mainstream.

### Supplementary Information

Supplementary Information.

## Data Availability

All data generated or analysed during this study are included in this published article [and its supplementary information files].
