# Supplementary material for: The improvement of block chain technology simulation in supply chain management (case study: pesticide company)
Source: Sci Rep. 2024 Feb 15;14:3784. doi: 10.1038/s41598-024-53694-w (PMC10869690; doi:10.1038/s41598-024-53694-w)
Supplement: Supplementary file 1 — Supplementary Information. [file 41598_2024_53694_MOESM1_ESM.pdf]

Table 1 Pestiside Product

Insektisida	Herbisida	Fungisida	Rodentisida	Lainnya
Mospilan 30 EC	Win 20 WG	Captive 200 SC	Ratgone 0,005 BB	Agrimore – Even
Ultimax 550 EC	Win 10 WP	Inari 72,5 WP		Agrimore – K
Tenchu 20 SG	Etron 670 EC	Nebijin 0,3 DP		Agrimore – N
Spontan 400 SL	Crash 480 SL	Blast 200 SC		Agrimore – P
Sevin 85 SP	Breeze 270 SL	Bazoka 80 WP		Cluster
Panzer 290 SL	Aladin 865 SL			Snaildown 250 EC
Maxima 68 WP				
Abuki 50 SL				

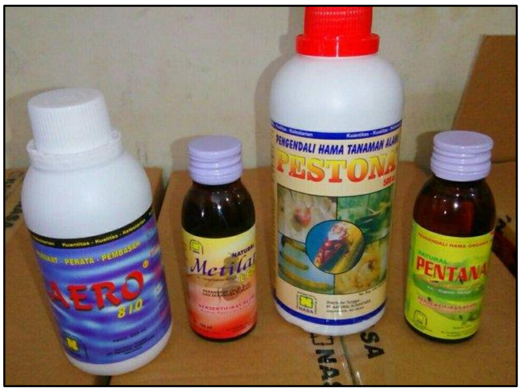

Figure. 1 Example of Pestiside Product

Tabel 2 Pestiside Production year 2013-2017

(Source: Company XXX year 2018)

Jenis Pestisida	Jumlah Produksi (Kg-Liter)				
	2013	2014	2015	2016	2017
Insektisida	1.825.980	1.912.174	1.132.944	1.685.397	2.079.481
Herbisida	1.076.560	1.907.480	1.837.331	1.734.468	1.612.778
Fungisida	385.786	438.724	280.988	537.466	528.962
Rodentisida	381.543	776.894	366.876	454.225	610.171

Tabel 3 Pestisidae Sales year 2013-2017

(Source: Pestiside company year 2018)

Jenis Pestisida	Jumlah Penjualan (Kg-Liter)				
	2013	2014	2015	2016	2017
Insektisida	1.580.354	1.644.485	1.012.493	1.685.420	2.097.595
Herbisida	1.074.656	1.828.948	1.801.355	1.722.288	1.662.766
Fungisida	319.216	386.944	261.610	540.332	553.775
Rodentisida	376.218	642.355	341.465	453.973	608.031

Table 4. ARP Value for Risk Agent

(source: Agusti Annisa Fany, 2020)

**Sort**	**Code**	**Risk Agent**	**ARP Value**
1.	A8	Damage to production tools and machines occurs	3.560
2.	A12	Line Stop	2.763
3.	A6	Technical Problem	1.848
4.	A4	Unqualified Material	1.632
5.	A5	Unqualified labor	1.515
6.	A24	Unstandardized Finished product	1.410
7.	A9	Lack of Maintenance	1.284
8.	A3	raw materials are not available	1.278
9.	A10	Human Error of Labor	1.124

Sort	Code	Risk Agent	ARP Value
10.	A11	Ignorance of work procedures	1.075
11.	A1	Error in Forecasting Calculation	1.068
12.	A16	Dependence on certain raw material suppliers	560
13.	A2	Emergency Order	532
14.	A17	Lack of Labor	351
15.	A14	Long Lead time	324
16.	A15	Damage material WIP	296
17.	A19	system reliability	288
18.	A21	There are limitations to the transportation fleet	276
19.	A20	Limited stock of finished products in warehouses/distribution centers	188
20.	A7	High production target	135
21.	A13	Long lead time from Abroad Supplier	106
22.	A22	Environment condition	88
23.	A18	Inefficient production	83
24.	A23	Stock of materials is piling up	24

Table 5. Risk Agent Classification

(source: Agusti Annisa Fany, 2020)

No	Code	ARP Value	Total Cum ARP	% Total Cum ARP	ABC Classification of Risk
1.	A8	3.560	3.560	16,32	A (High Risk)
2.	A12	2.763	6.323	28,99	
3.	A6	1.848	8.171	37,47	
4.	A4	1.632	9.803	44,95	
5.	A5	1.515	11.318	51,90	
6.	A24	1.410	12.728	58,63	
7.	A9	1.284	14.012	64,25	
8.	A3	1.278	15.290	70,11	

No	Code	ARP Value	Total Cum ARP	% Total Cum ARP	ABC Classification of Risk
9.	A10	1.124	16.414	75,27	
10.	A11	1.075	17.489	80,20	
11.	A1	1.068	18.557	85,09	B (Medium Risk)
12.	A16	560	19.117	87,66	
13.	A2	532	19.649	90,10	
14.	A17	351	20.000	91,71	
15.	A14	324	20.324	93,20	
16.	A15	296	20.620	94,55	
17.	A19	288	20.908	95,87	C (Low Risk)
18.	A21	276	21.184	97,14	
19.	A20	188	21.372	98,00	
20.	A7	135	21.507	98,62	
21.	A13	106	21.613	99,11	
22.	A22	88	21.701	99,51	
23.	A18	83	21.784	99,89	
24.	A23	24	21.808	100,00	

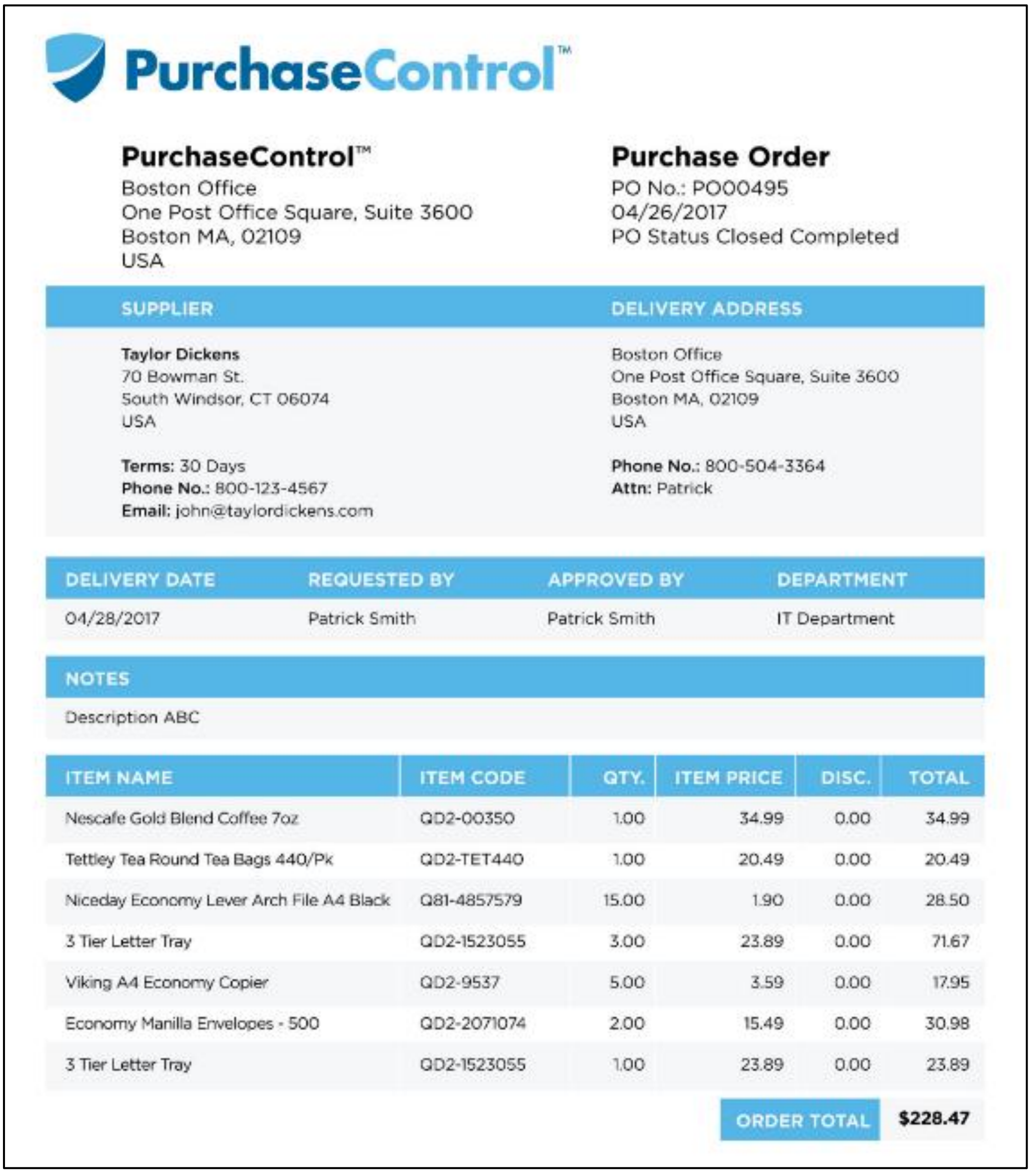

PurchaseControl™
Boston Office
One Post Office Square, Suite 3600
Boston MA, 02109
USA

Purchase Order
PO No.: PO00495
04/26/2017
PO Status Closed Completed

SUPPLIER	DELIVERY ADDRESS
**Taylor Dickens**<br>70 Bowman St.<br>South Windsor, CT 06074<br>USA<br><br>**Terms:** 30 Days<br>**Phone No.:** 800-123-4567<br>**Email:** john@taylordickens.com	Boston Office<br>One Post Office Square, Suite 3600<br>Boston MA, 02109<br>USA<br><br>**Phone No.:** 800-504-3364<br>**Attn:** Patrick

DELIVERY DATE	REQUESTED BY	APPROVED BY	DEPARTMENT
04/28/2017	Patrick Smith	Patrick Smith	IT Department

NOTES
Description ABC

ITEM NAME	ITEM CODE	QTY.	ITEM PRICE	DISC.	TOTAL
Nescafe Gold Blend Coffee 7oz	QD2-00350	1.00	34.99	0.00	34.99
Tettley Tea Round Tea Bags 440/Pk	QD2-TET440	1.00	20.49	0.00	20.49
Niceday Economy Lever Arch File A4 Black	Q81-4857579	15.00	1.90	0.00	28.50
3 Tier Letter Tray	QD2-1523055	3.00	23.89	0.00	71.67
Viking A4 Economy Copier	QD2-9537	5.00	3.59	0.00	17.95
Economy Manilla Envelopes - 500	QD2-2071074	2.00	15.49	0.00	30.98
3 Tier Letter Tray	QD2-1523055	1.00	23.89	0.00	23.89
				ORDER TOTAL	**$228.47**

Figure 2 Example of *Purchase Order Document*

(Sumber Gambar: https://www.purchasecontrol.com/wp-content/uploads/2019/01/what-is-a-purchase-order-example.jpg)

PT CITRA GO ESTATE

Email : citrago3state@gmail.com Telp : (024) 2409285
Jl. Simongan No.39
Semarang-Jawa Tengah

SURAT PERJANJIAN KERJASAMA

PELAKSANAAN PEKERJAAN PEMASOKAN BAHAN PENGURUGAN REKLAMASI

Yang bertandatangan di bawahini

Nama : Riadi Prabowo, S.T., M.T

Alamat : Jalan Simongan Nomor 39, Semarang

Jabatan : Direktur Utama PT Citra Go Estate

Selanjutnya disebut sebagai PIHAK KESATU

Nama : Ririyani Nurhawary, S.T

Alamat : Jalan Menoreh Raya Nomor 32, Semarang

Jabatan : Direktur CV Astra Material

Selanjutnya disebut sebagai PIHAK KEDUA

KEDUA BELAH PIHAK sepakat melaksanakan kerjasama untuk melakukan pekerjaan pemasokan tanah *alluvial* dan batuan *andesit* dalam proyek reklamasi dengan sumber dana dari PT Dyah Intiland Development. Ketentuan dan tata cara yang diatur sebagai berikut:

Pasal 1

Ketentuan Umum

1 Kata-kata dan ungkapan-ungkapan dalam surat perjanjian ini mempunyai arti yang sama sebagaimana yang dituangkan di dalam syarat-syarat surat perjanjian di bawah ini.

Figure 3 Reference Document as Contract Simulation Format

(Source: https://lezgetreal.com/contoh-surat-perjanjian-kerjasama-supplier/#!)

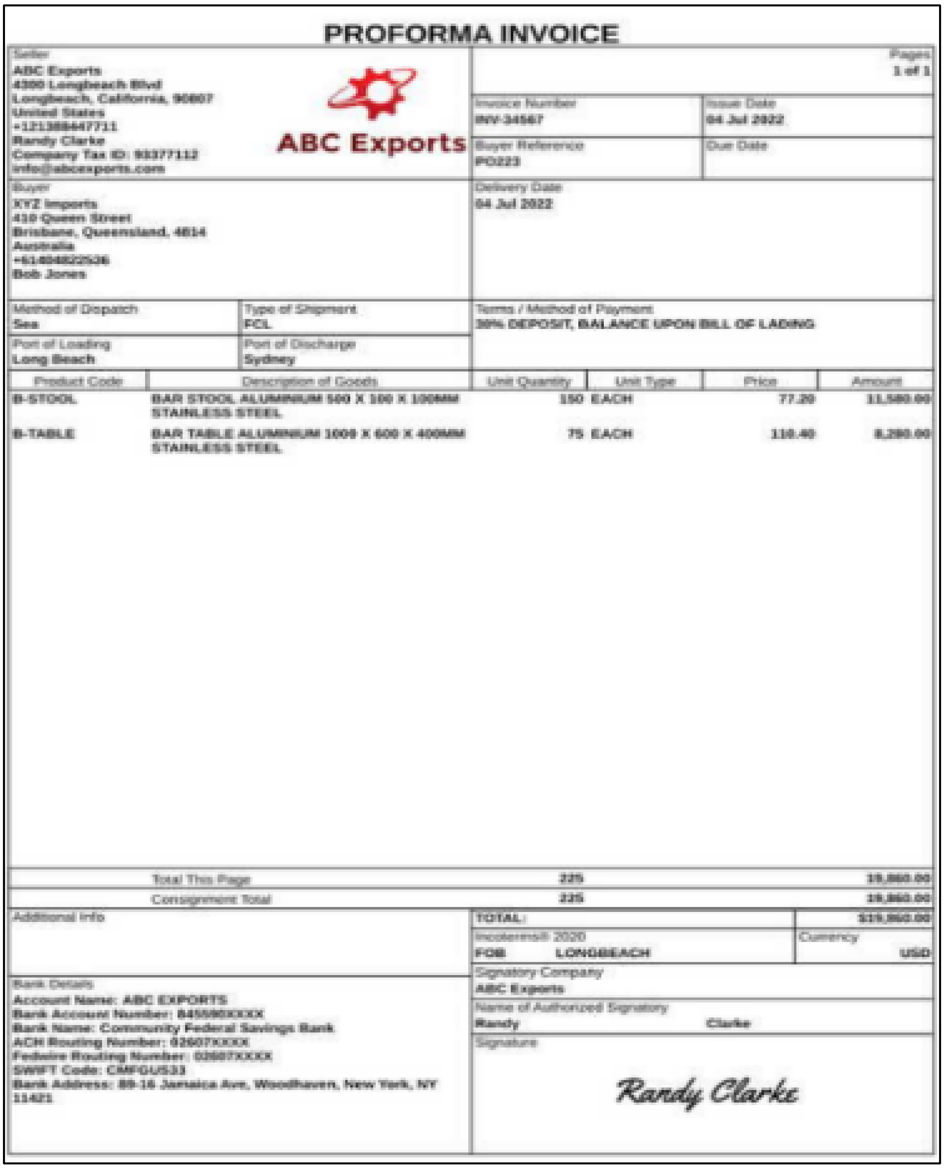

PROFORMA INVOICE

Seller<br>ABC Exporters<br>4300 Longbeach Blvd<br>Longbeach, California, 90807<br>United States<br>+12138884477711<br>Randy Clarke<br>Company Tax ID: 93377112<br>info@abcexports.com	ABC Exports	Pages<br>1 of 1	
		Invoice Number<br>INV-34567	Issue Date<br>04 Jul 2022
		Buyer Reference<br>PO223	Due Date
Buyer<br>XYZ Imports<br>410 Queen Street<br>Brisbane, Queensland, 4814<br>Australia<br>+61404822526<br>Bob Jones		Delivery Date<br>04 Jul 2022	
Method of Dispatch<br>Sea	Type of Shipment<br>FCL	Terms / Method of Payment<br>30% DEPOSIT, BALANCE UPON BILL OF LADING	
Port of Loading<br>Long Beach	Port of Discharge<br>Sydney		

Product Code	Description of Goods	Unit Quantity	Unit Type	Price	Amount
B-STOOL	BAR STOOL ALUMINIUM 500 X 100 X 100MM STAINLESS STEEL	150	EACH	77.20	11,580.00
B-TABLE	BAR TABLE ALUMINIUM 1000 X 600 X 400MM STAINLESS STEEL	75	EACH	110.40	8,280.00
	Total This Page	225			19,860.00
	Consignment Total	225			19,860.00

Additional Info	TOTAL:	$19,860.00
	Incoterms[illegible] 2020<br>FOB LONGBEACH	Currency<br>USD
Bank Details<br>Account Name: ABC EXPORTS<br>Bank Account Number: 845590XXXX<br>Bank Name: Community Federal Savings Bank<br>ACH Routing Number: 02607XXXX<br>Fedwire Routing Number: 02607XXXX<br>SWIFT Code: CMFGUS33<br>Bank Address: 89-16 Jamaica Ave, Woodhaven, New York, NY 11421	Signatory Company<br>ABC Exports	
	Name of Authorized Signatory<br>Randy Clarke	
	Signature<br>Randy Clarke	

Figure 4 Reference Document as Proforma Invoice Simulation Format

(Source: https://incodocs.com/template/proforma_invoice)

Boston Office
One Post Office Square, Suite 3600
Boston MA, 02108
USA

PO No: PO00495
04/26/2017
PO Status Closed Completed

SUPPLIER	DELIVERY ADDRESS
**Taylor Dickens**<br>70 Bowman St.<br>South Windsor, CT 06074<br>USA<br><br>**Terms:** 30 Days<br>**Phone No:** 800-123-4567<br>**Email:** john@taylordickens.com	**Boston Office**<br>One Post Office Square, Suite 3600<br>Boston MA, 02109<br>USA<br><br>**Phone No:** 800-123-4567<br>**Attn:** Patrick

DELIVERY DATE	REQUESTED BY	APPROVED BY	DEPARTMENT
04/28/2017	Patrick Smith	John Smith	IT Department

NOTES
Description ABC

ITEM NAME	ITEM CODE	QTY	ITEM PRICE	DISC.	TOTAL
Nescafe Gold Blend Coffee 7oz	[illegible]	1.00	34.99	0.00	34.99
Tetley Tea Round Tea Bags 440Pk	[illegible]	1.00	20.49	0.00	20.49
[illegible] Economy Lever Arch File A4 Black	[illegible]	15.00	1.90	0.00	28.50
5 Tier Letter Tray	[illegible]	[illegible]	[illegible]	0.00	[illegible]
Viking A4 Economy Copier	[illegible]	5.00	3.59	0.00	17.95
Economy Manila Envelopes - 500	[illegible]	2.00	15.49	0.00	30.98
5 Tier Letter Tray	[illegible]	1.00	[illegible]	0.00	[illegible]
				ORDER TOTAL	$228.47

Figure 5 Document Reference as Company Purchase Order Simulation Format

(Source: https://planergy.com/)

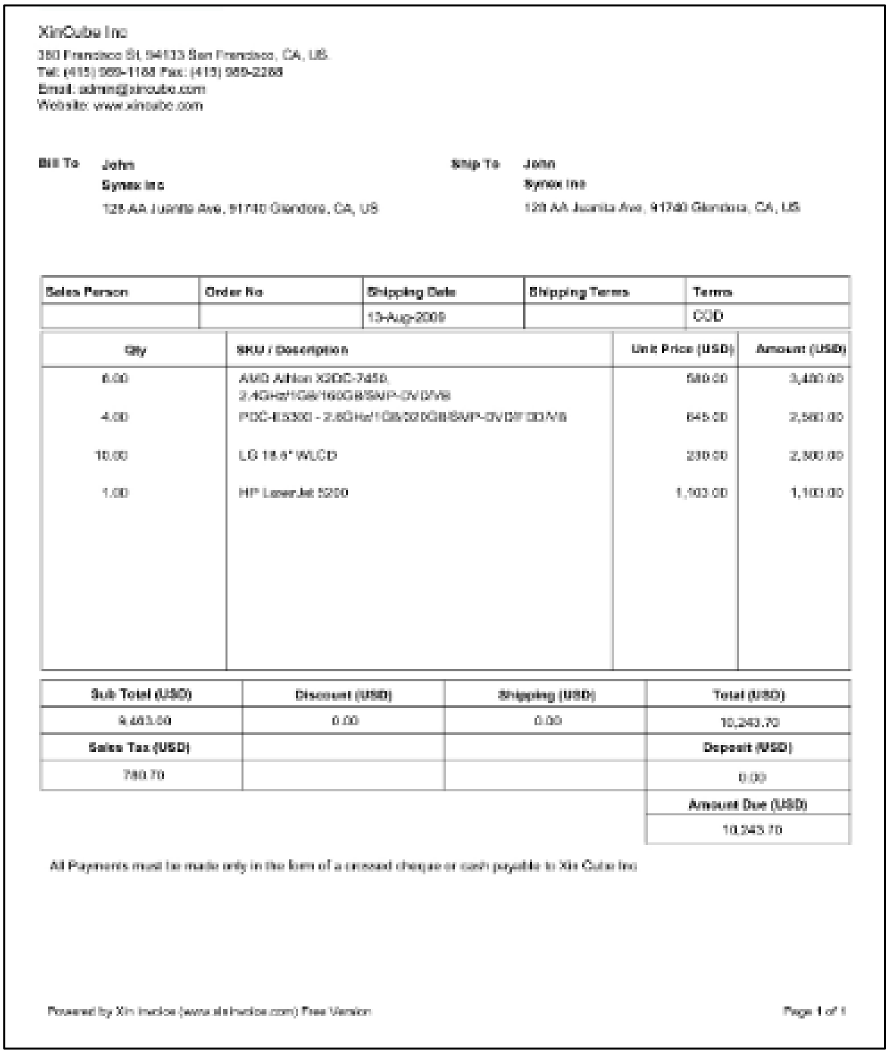

XinCube Inc
350 Francisco St, 94133 San Francisco, CA, US
Tel: (415) 969-1168 Fax: (415) 969-2268
Email: admin@xincube.com
Website: www.xincube.com

Bill To **John**
Synex Inc
128 AA Juanita Ave, 91740 Glendora, CA, US

Ship To **John**
Synex Inc
128 AA Juanita Ave, 91740 Glendora, CA, US

Sales Person	Order No	Shipping Date	Shipping Terms	Terms
		13-Aug-2009		COD

Qty	SKU / Description	Unit Price (USD)	Amount (USD)
6.00	AMD Athlon X2DC-7450, 2.4GHz/1GB/160GB/SMP-DVD/VB	580.00	3,480.00
4.00	PDC-E5300 - 2.6GHz/1GB/320GB/SMP-DVDRW/VB	645.00	2,580.00
10.00	LG 18.5" WLCD	230.00	2,300.00
1.00	HP LaserJet 5200	1,100.00	1,100.00

Sub Total (USD)	Discount (USD)	Shipping (USD)	Total (USD)
9,463.00	0.00	0.00	10,243.70
**Sales Tax (USD)**			**Deposit (USD)**
780.70			0.00
			**Amount Due (USD)**
			10,243.70

All Payments must be made only in the form of a crossed cheque or cash payable to Xin Cube Inc

Powered by Xin Invoice ([illegible]) Free Version   Page 1 of 1

Figure 6 Reference Document as Supplier Invoice Simulation Format

(Source: https://simpleinvoice17.net/)

ABC Company

No. 24, Ghandhi Nagar
Bangalore

Payment Advice

M/s. Rajesh K Date : **13-Feb-2014**

#43, MCB Building
Cauvery Building
3rd Cross, 5th Main, Jayanagar

Dear Sir/Madam,

Please find below the payment details.

Bill Ref.	Bill Date	Amount
New Ref / Bill Apr/03a1	24-Sep-2013	3,600.0
	**Nett Amount**	**In₹ 3,600.0**

Payment Details				
**Payment Mode**	**Transferred to**	**Instrument Details**	**Issued From**	**Amount**
Inter Bank Transfer	A/c.No.: 0096927712122112<br>Bank: Indian Overseas Bank<br>IFSC :IOBA0009090	No.: IOBA121234554321<br>Dt: 24-Sep-2013	HDFC Bank	3,600.00
			**Total**	**₹ 3,600.00**

Kindly acknowledge the receipt.

Thanking You

Authorised Signatory Receiver's Signature

Figure 7 Reference Document as Payment Note Simulation Format

(Source : https://resources.tallysolutions.com/)

Boston Office
One Post Office Square, Suite 3600
Boston MA, 02109
USA

PO No: PO00495
04/26/2017
PO Status Closed Completed

SUPPLIER	DELIVERY ADDRESS
**Taylor Dickens**<br>70 Bowman St.<br>South Windsor, CT 06074<br>USA<br><br>**Terms:** 30 Days<br>**Phone No:** 800-123-4567<br>**Email:** john@taylorcdickens.com	**Boston Office**<br>One Post Office Square, Suite 3600<br>Boston MA, 02109<br>USA<br><br>**Phone No:** 800-123-4567<br>**Attn:** Patrick

DELIVERY DATE	REQUESTED BY	APPROVED BY	DEPARTMENT
04/28/2017	Patrick Smith	John Smith	IT Department

NOTES
Description ABC

ITEM NAME	ITEM CODE	QTY	ITEM PRICE	DISC.	TOTAL
Nescafe Gold Blend Coffee 7oz	QD2-00350	1.00	34.99	0.00	34.99
Tettley Tea Round Tea Bags 440/Pk	QD2-TET440	1.00	20.49	0.00	20.49
Niceday Economy Lever Arch File A4 Black	Q81-4857579	15.00	1.80	0.00	28.50
3 Tier Letter Tray	QD2-1523055	3.00	23.89	0.00	71.67
Viking A4 Economy Copier	QD2-8537	5.00	3.59	0.00	17.95
Economy Manila Envelopes - 500	QD2-2071074	2.00	15.49	0.00	30.98
3 Tier Letter Tray	QD2-1523055	1.00	23.89	0.00	23.89
				ORDER TOTAL	$228.47

Figure 8 Reference Document as Format for Buyer's Purchase Order Simulation

(Sumber: https://planergy.com/)

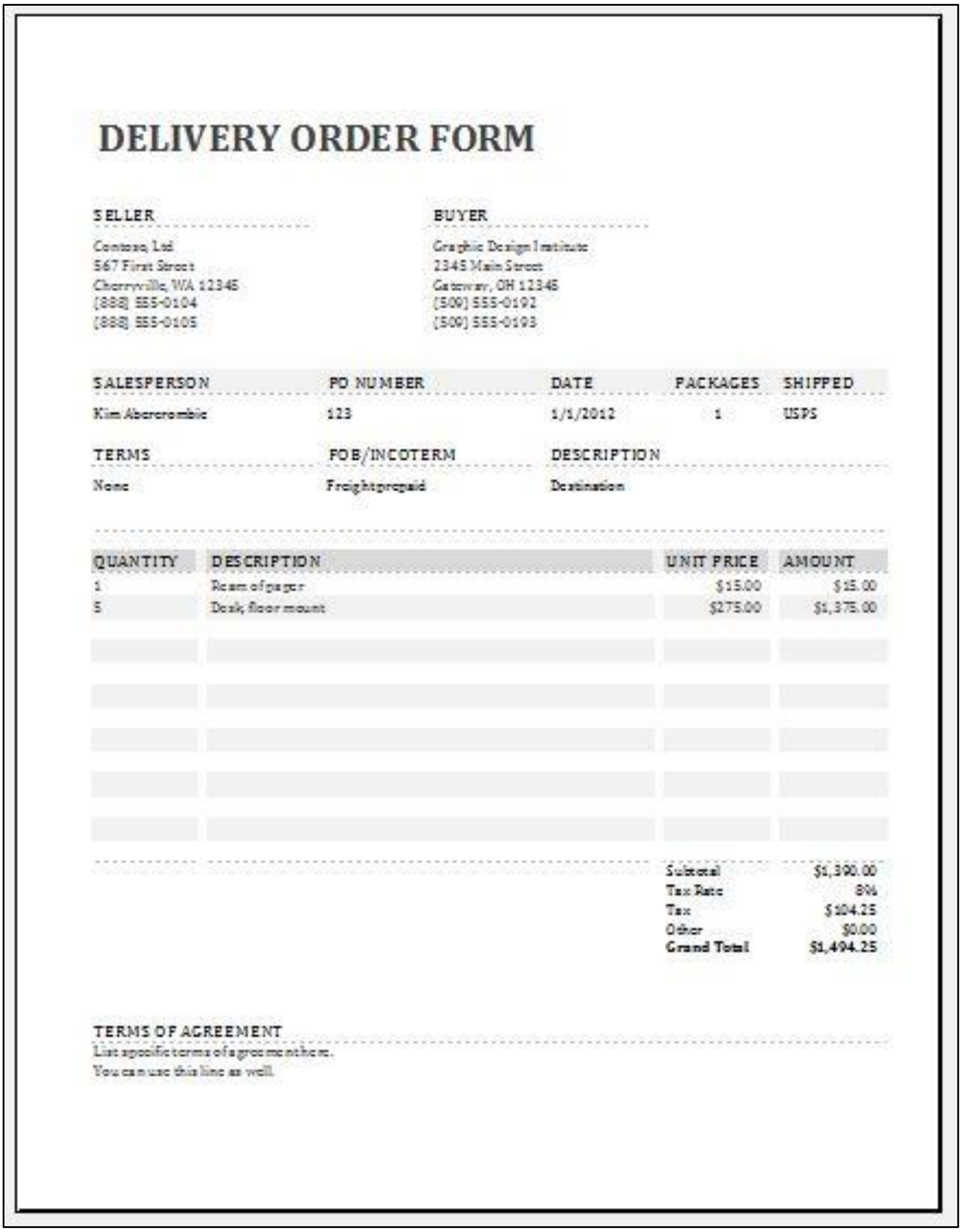

DELIVERY ORDER FORM

SELLER	BUYER
Contoso, Ltd	Graphic Design Institute
567 First Street	2345 Main Street
Cherryville, WA 12345	Gateway, OH 12345
(888) 555-0104	(509) 555-0192
(888) 555-0105	(509) 555-0193

SALESPERSON	PO NUMBER	DATE	PACKAGES	SHIPPED
Kim Abercrombie	123	1/1/2012	1	USPS

TERMS	FOB/INCOTERM	DESCRIPTION
None	Freight prepaid	Destination

QUANTITY	DESCRIPTION	UNIT PRICE	AMOUNT
1	Ream of paper	$15.00	$15.00
5	Desk, floor mount	$275.00	$1,375.00
		Subtotal	$1,390.00
		Tax Rate	8%
		Tax	$104.25
		Other	$0.00
		**Grand Total**	**$1,494.25**

TERMS OF AGREEMENT

List specific terms of agreement here.

You can use this line as well.

Figure 9 Reference Document as Delivery Order Simulation Format

(Source: https://www.wordexceltemplates.com/)

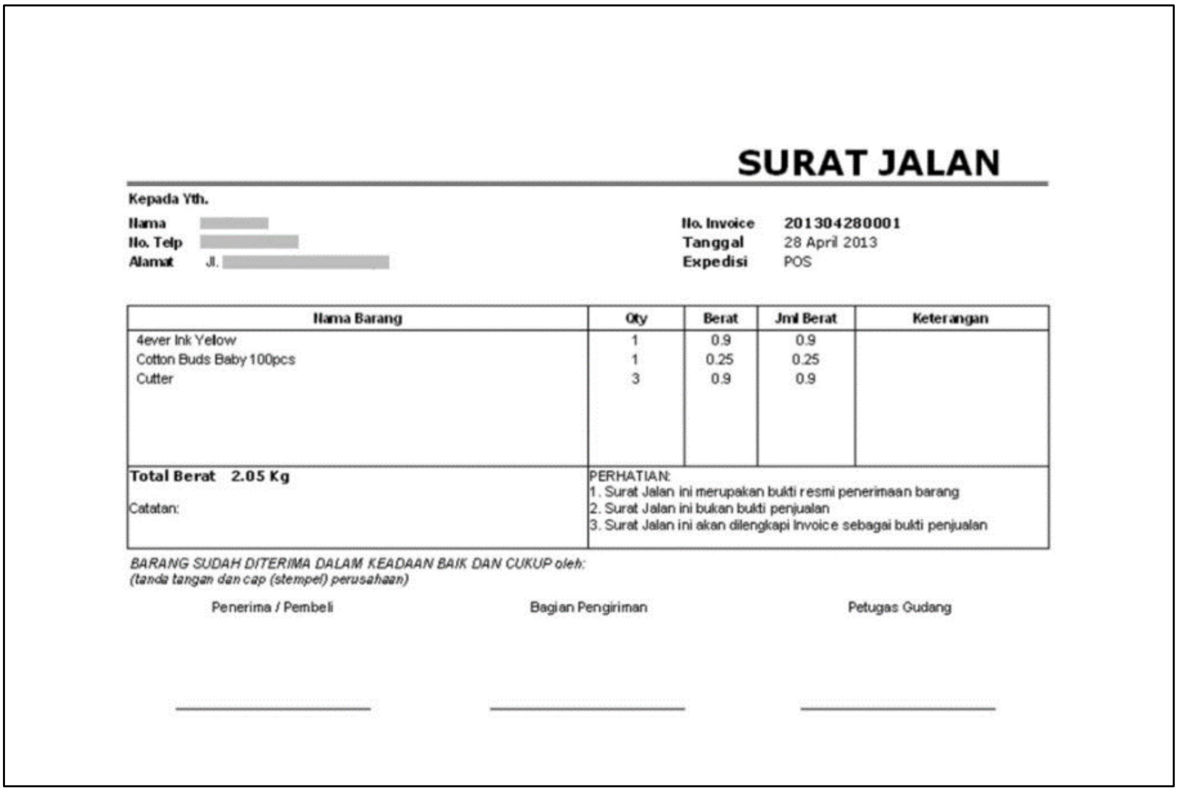

SURAT JALAN

Kepada Yth.

Nama [redacted]
No. Telp [redacted]
Alamat Jl. [redacted]

No. Invoice **201304280001**
Tanggal 28 April 2013
Expedisi POS

Nama Barang	Qty	Berat	Jml Berat	Keterangan
4ever Ink Yellow	1	0.9	0.9	
Cotton Buds Baby 100pcs	1	0.25	0.25	
Cutter	3	0.9	0.9	

Total Berat 2.05 Kg

Catatan:

PERHATIAN:
1. Surat Jalan ini merupakan bukti resmi penerimaan barang
2. Surat Jalan ini bukan bukti penjualan
3. Surat Jalan ini akan dilengkapi Invoice sebagai bukti penjualan

BARANG SUDAH DITERIMA DALAM KEADAAN BAIK DAN CUKUP oleh:
(tanda tangan dan cap (stempel) perusahaan)

Penerima / Pembeli — Bagian Pengiriman — Petugas Gudang

Figure 10 Document Reference as Travel Document Simulation Format

(Source: https://kargo.tech/)

XinCube Inc
380 Francisco St, 94133 San Francisco, CA, US.
Tel: (415) 989-1188 Fax: (415) 989-2288
Email: admin@xincube.com
Website: www.xincube.com

Bill To **John**
Synex Inc
128 AA Juanita Ave, 91740 Glendora, CA, US

Ship To **John**
Synex Inc
128 AA Juanita Ave, 91740 Glendora, CA, US

Sales Person	Order No	Shipping Date	Shipping Terms	Terms
		13-Aug-2009		COD

Qty	SKU / Description	Unit Price (USD)	Amount (USD)
6.00	AMD Athlon X2DC-7450, 2.4GHz/1GB/160GB/SMP-DVD/VB	580.00	3,480.00
4.00	PDC-E5300 - 2.6GHz/1GB/320GB/SMP-DVD/FDD/VB	645.00	2,580.00
10.00	LG 18.5" WLCD	230.00	2,300.00
1.00	HP LaserJet 5200	1,103.00	1,103.00

Sub Total (USD)	Discount (USD)	Shipping (USD)	Total (USD)
9,463.00	0.00	0.00	10,243.70
**Sales Tax (USD)**			**Deposit (USD)**
780.70			0.00
			**Amount Due (USD)**
			10,243.70

All Payments must be made only in the form of a crossed cheque or cash payable to Xin Cube Inc

Powered by Xin Invoice (www.xininvoice.com) Free Version

Page 1 of 1

Figure 11

Document Reference as Company Invoice Simulation Format

(Sumber: https://simpleinvoice17.net/)

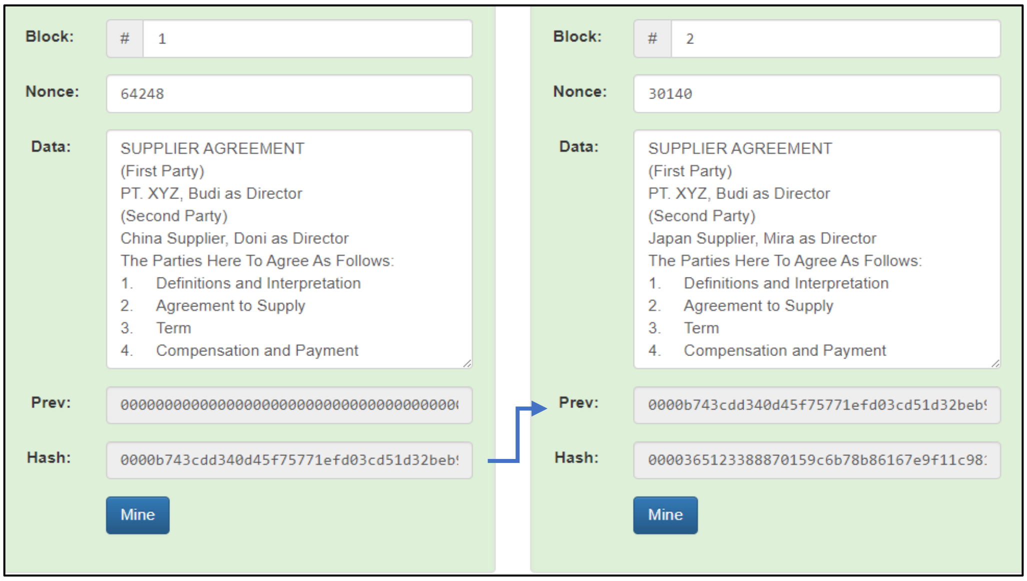

Figure 12 Contract Simulation Results in Block 1 and Block 2

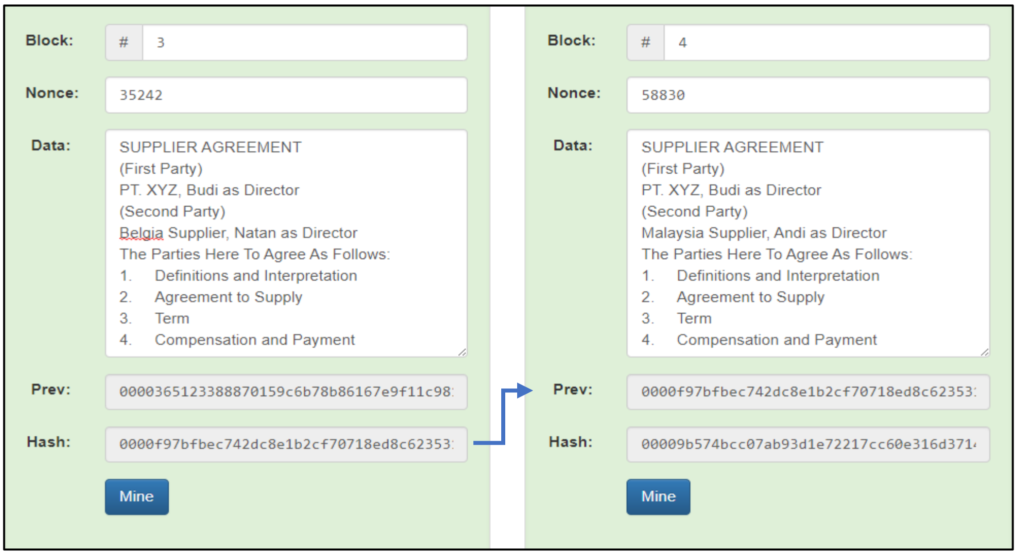

Figure 13 Contract Simulation Results in Block 3 and Block 4

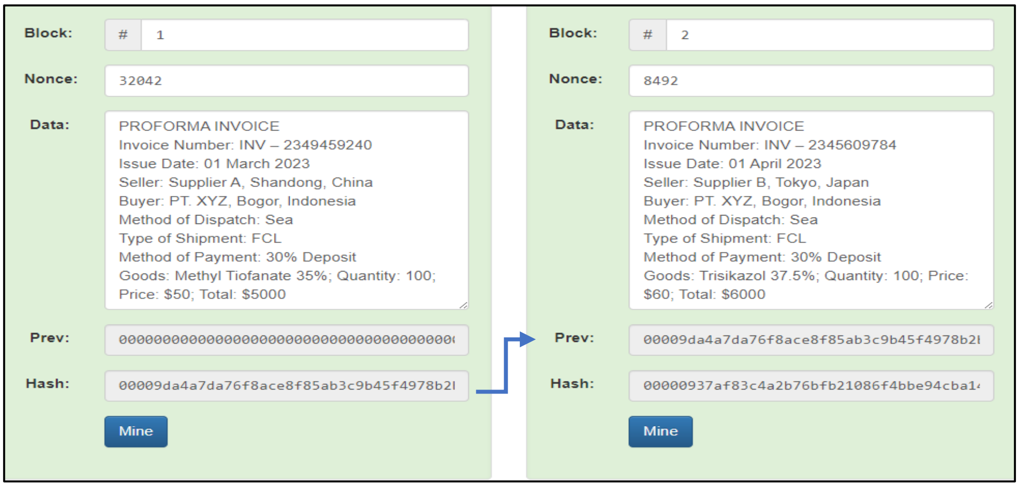

Figure 14 Proforma Invoice Simulation Results for Block 1 and Block 2

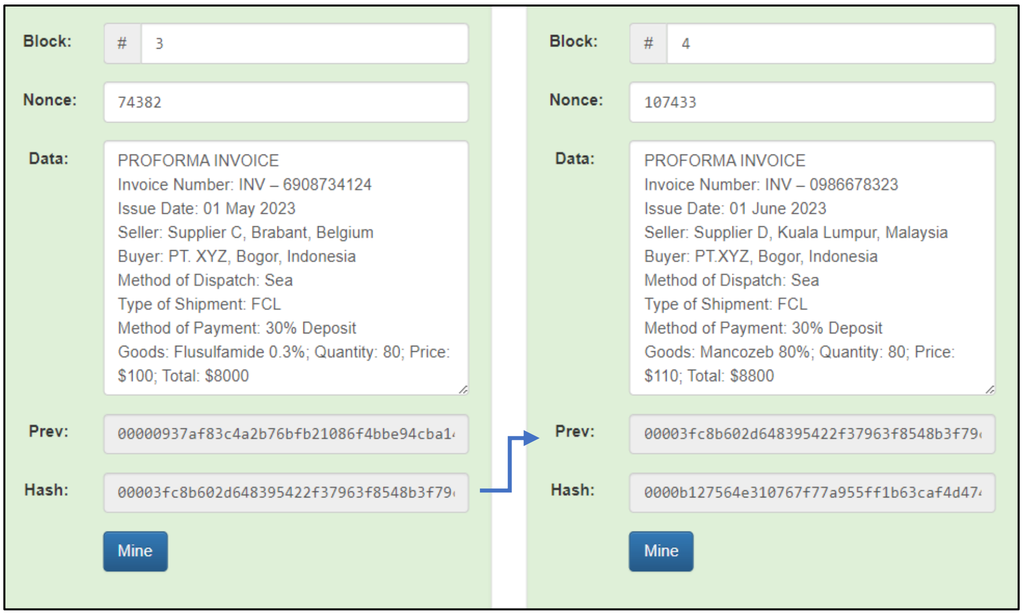

Figure 15 Proforma Invoice Simulation Results for Block 3 and Block 4

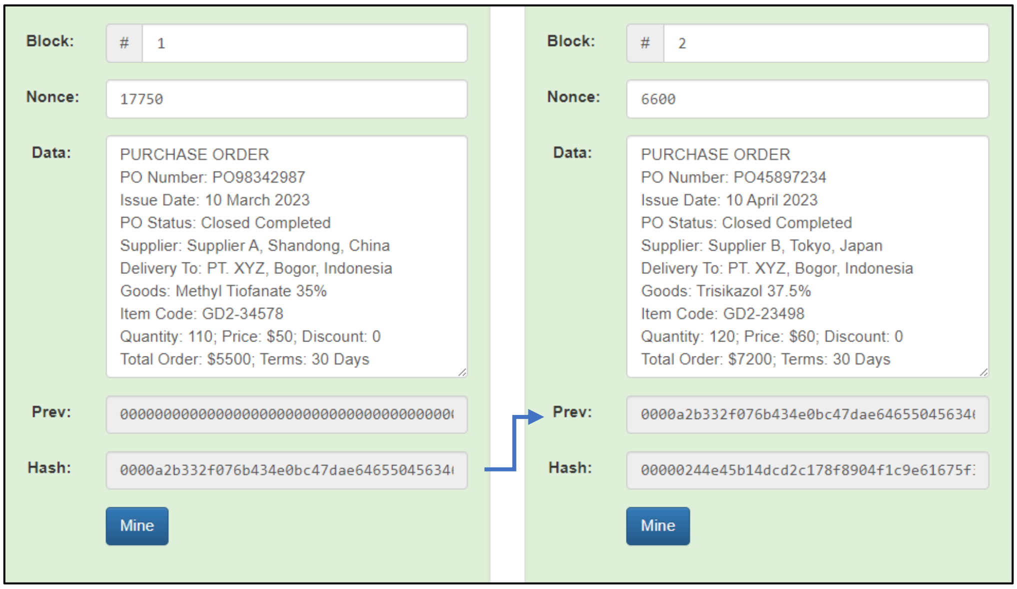

Figure 16

Simulation Results of Company Purchase Orders in Block 1 and Block 2

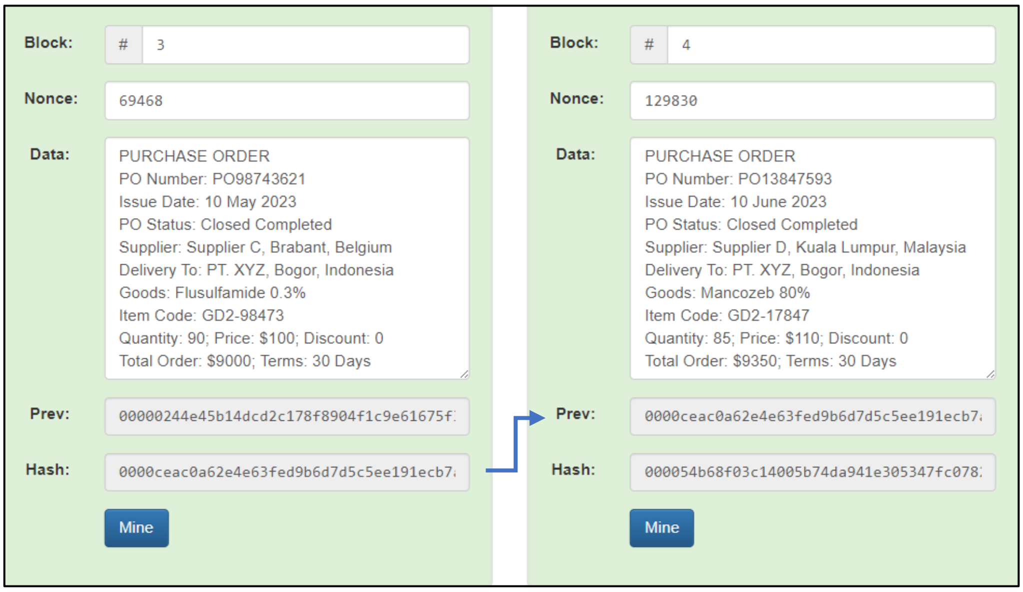

Figure 17

Simulation Results of Company Purchase Orders in Block 3 and Block 4

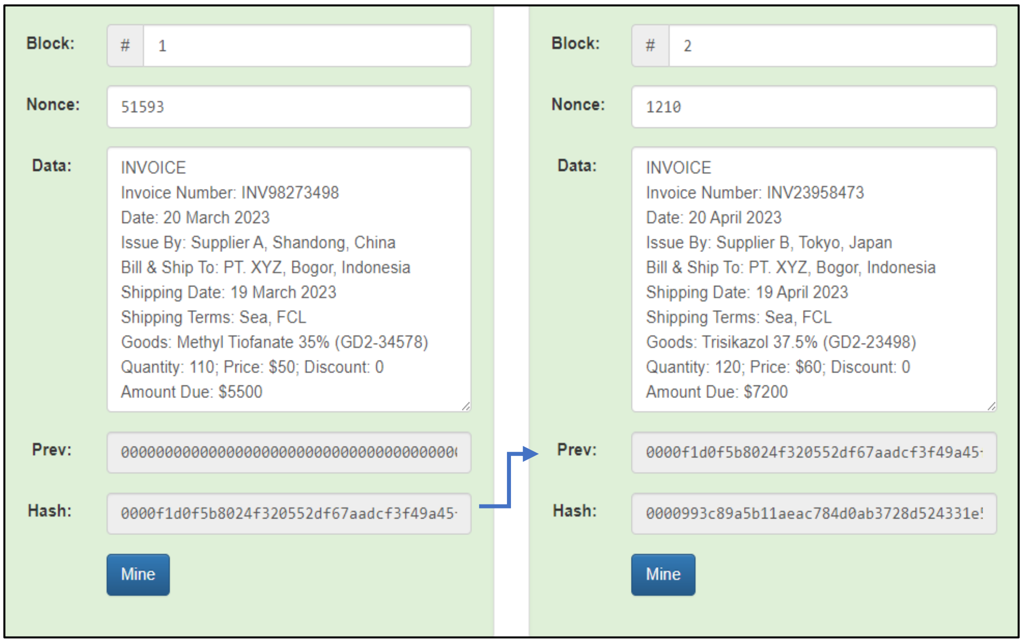

Figure 18 Supplier Invoice Simulation Results in Block 1 and Block 2

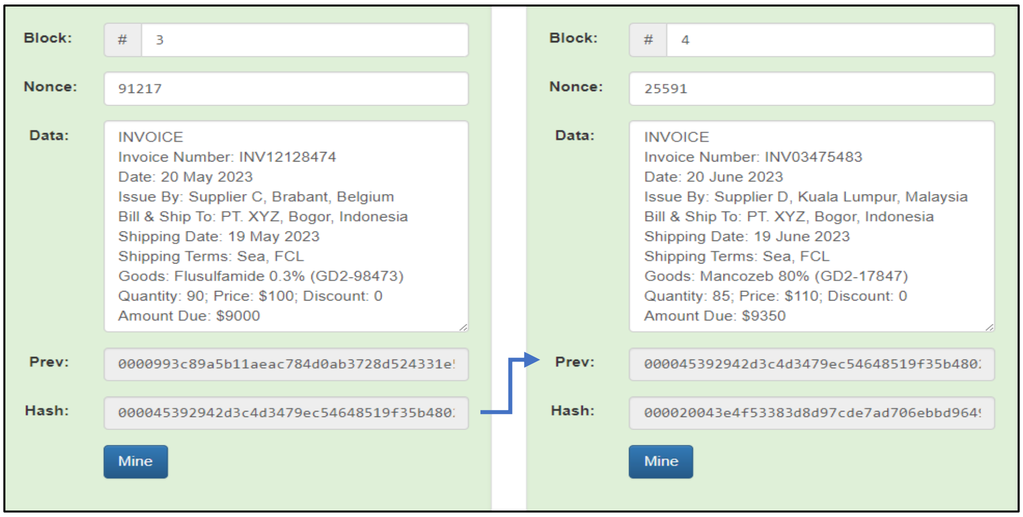

Figure 19 Supplier Invoice Simulation Results in Block 3 and Block 4

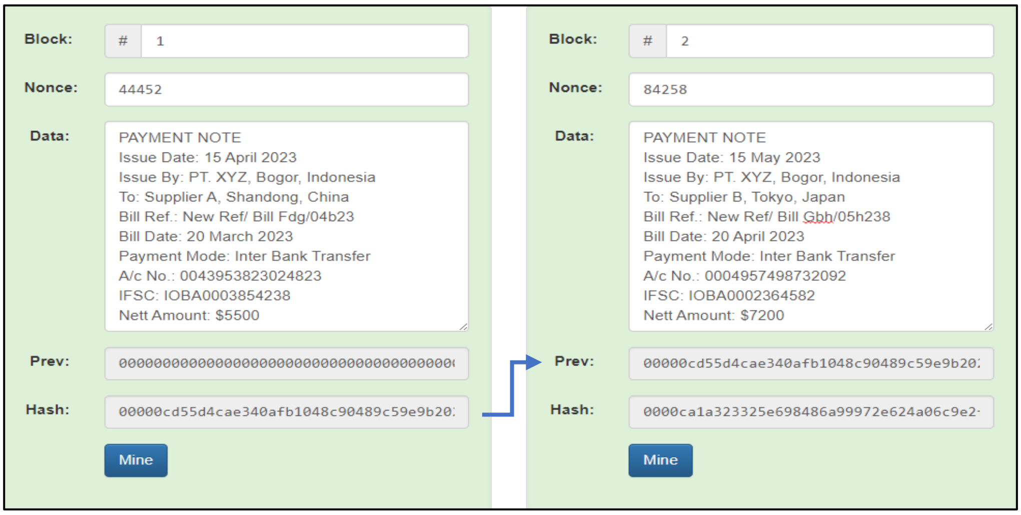

Figure 20 Payment Note Simulation Results in Block 1 and Block 2

Begitu pula dengan blok 3 dan blok 4 juga menjadikan *hash* pada blok sebelumnya menjadi *previous hash* pada blok tersebut. Blok 3 berhasil menghasilkan *hash* bernilai “00003667616e29278bcdee28e5eb3e0b89974d”. Blok 4 berhasil menghasilkan *hash* bernilai “0000698b8edbbc0439b507348d4764b93435b6”. Hasil simulasi blok 3 dan blok 4 dapat dilihat pada Gambar 4.29.

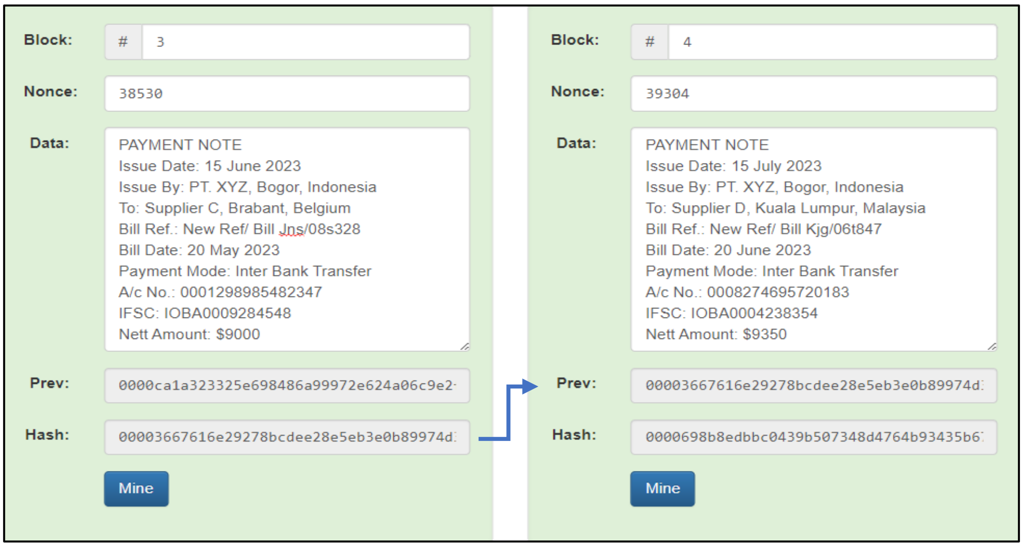

Figure 21 Payment Note Simulation Results in Block 3 and Block 4

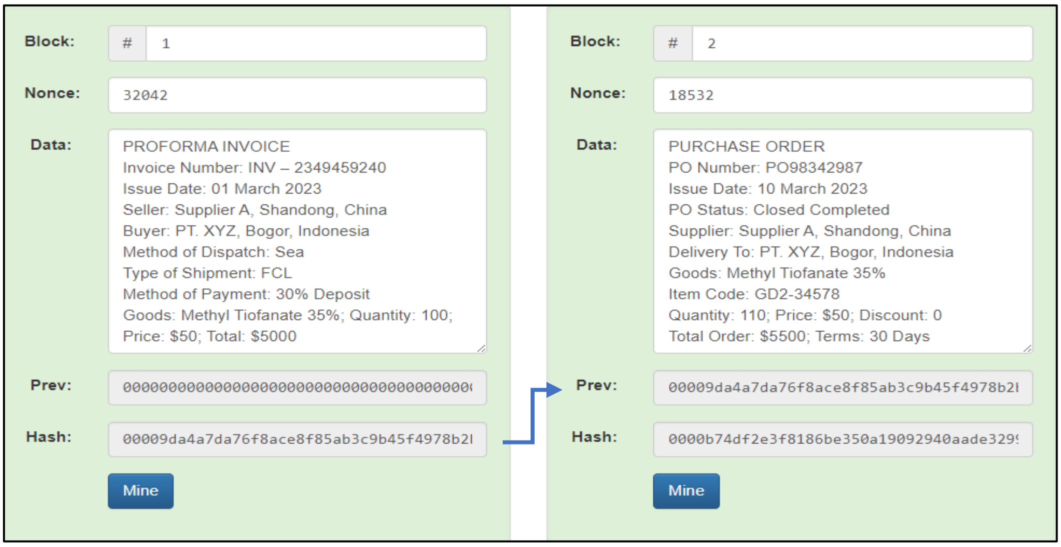

Figure 22 Simulation Results According to the Sequence of Raw Material Procurement Process in Block 1 and Block 2

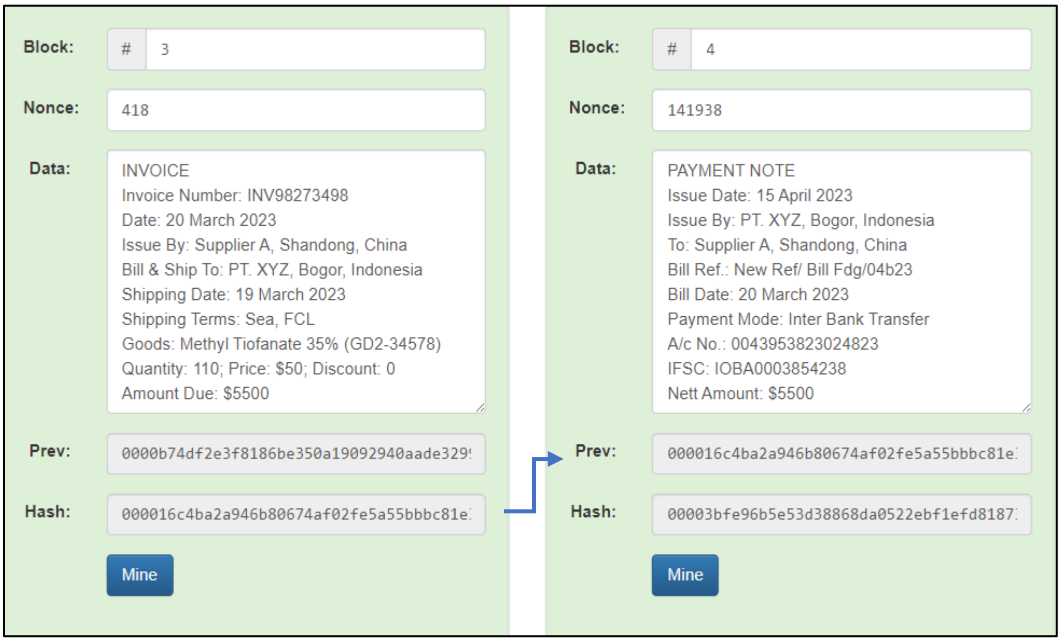

Figure 23 Simulation Results According to the Sequence of Raw Material Procurement Process in Block 3 and Block 4

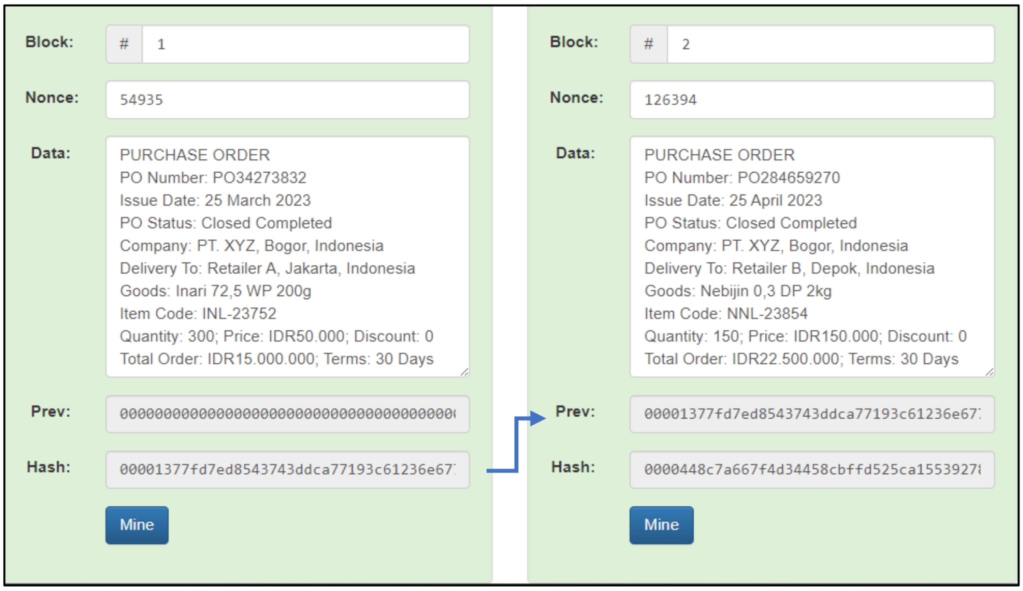

Figure 24 Simulation Results of Buyer's Purchase Orders in Block 1 and Block 2

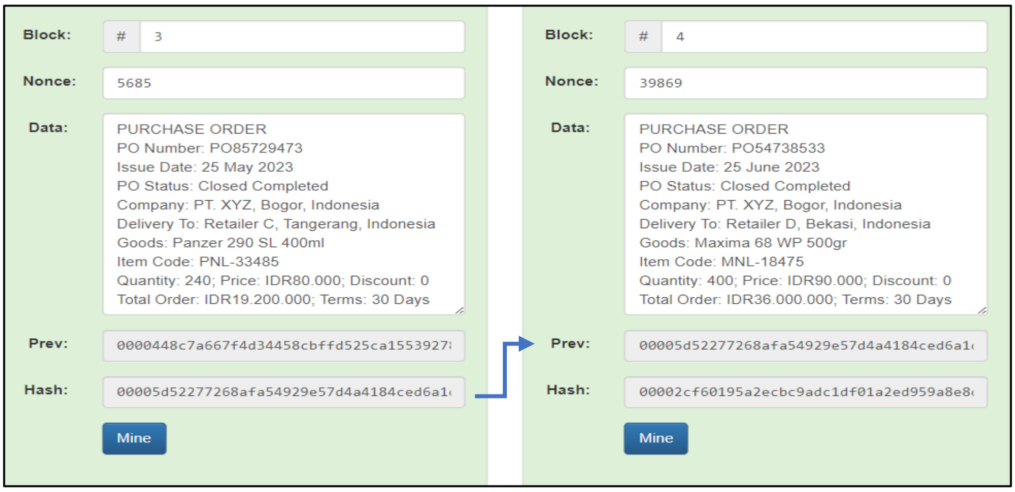

Figure 25 Simulation Results of Buyer's Purchase Orders in Block 3 and Block 4

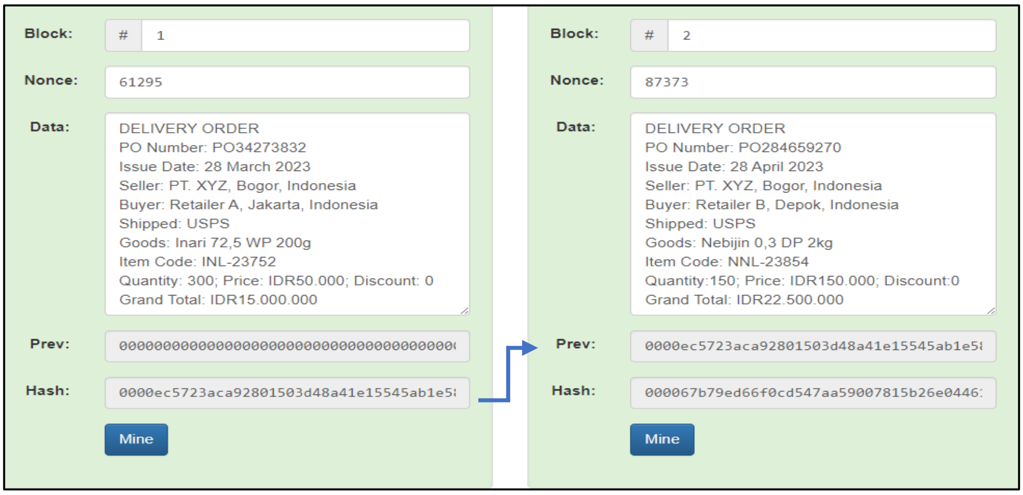

Figure 26 Delivery Order Simulation Results in Block 1 and Block 2

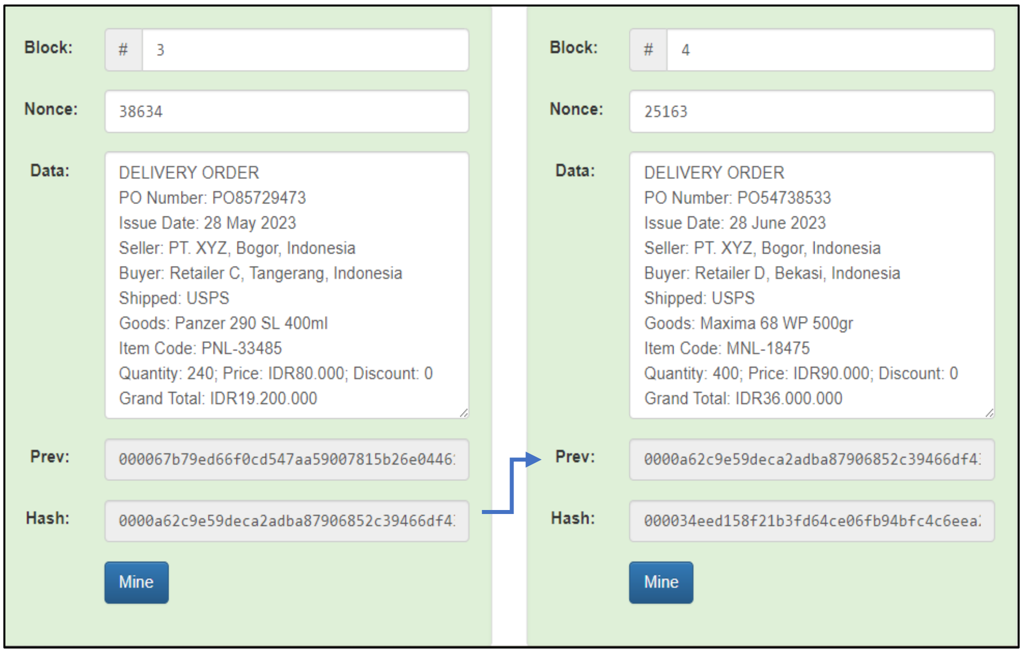

Figure 27 Delivery Order Simulation Results in Block 3 and Block 4

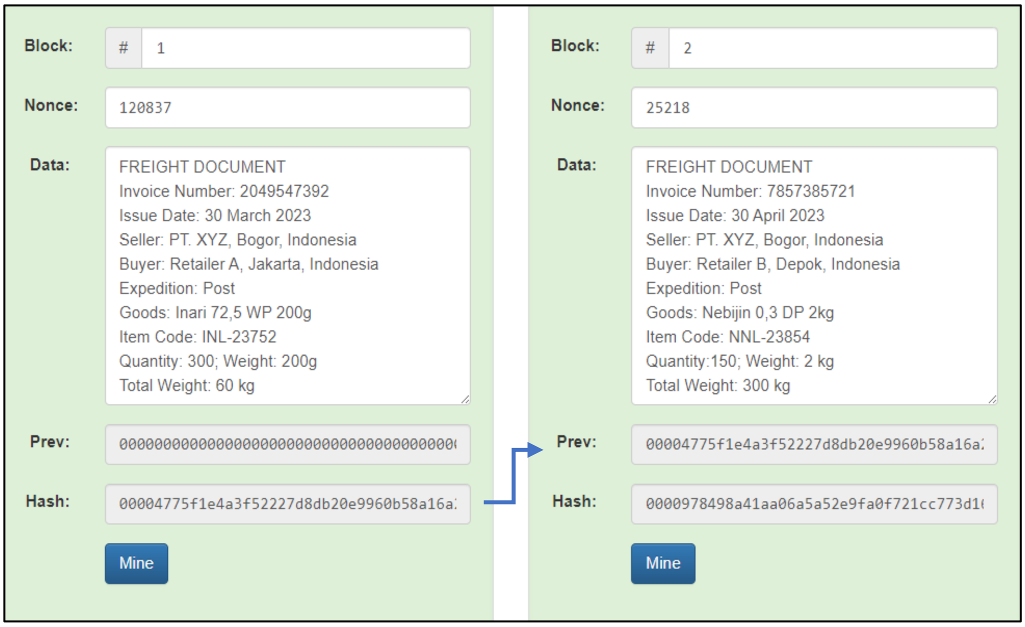

Figure 28 Simulation Results of Travel Documents in Block 1 and Block 2

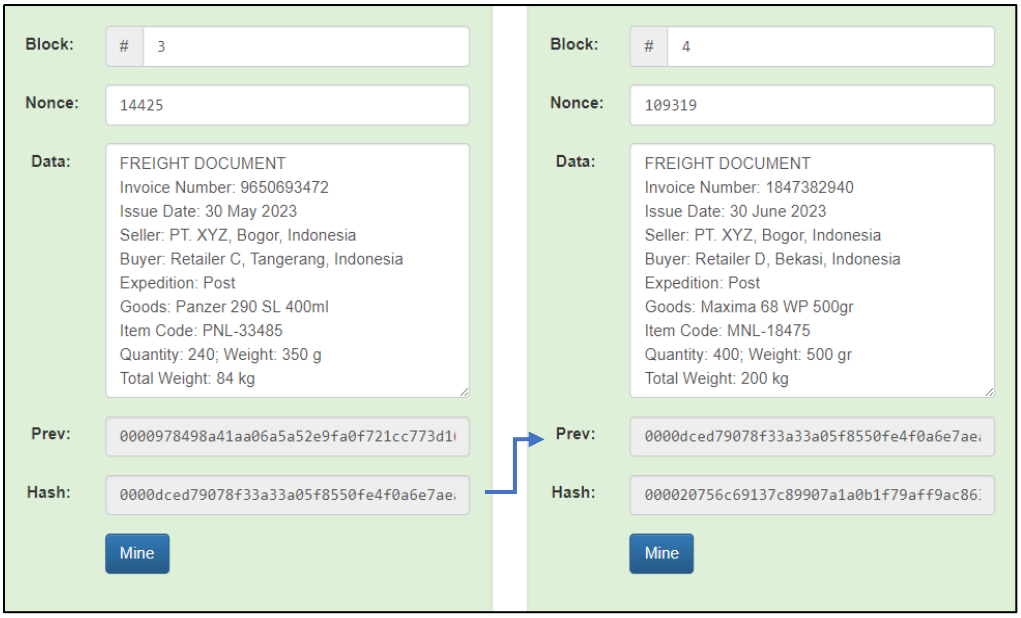

Figure 29 Simulation Results of Travel Documents in Block 3 and Block 4

.

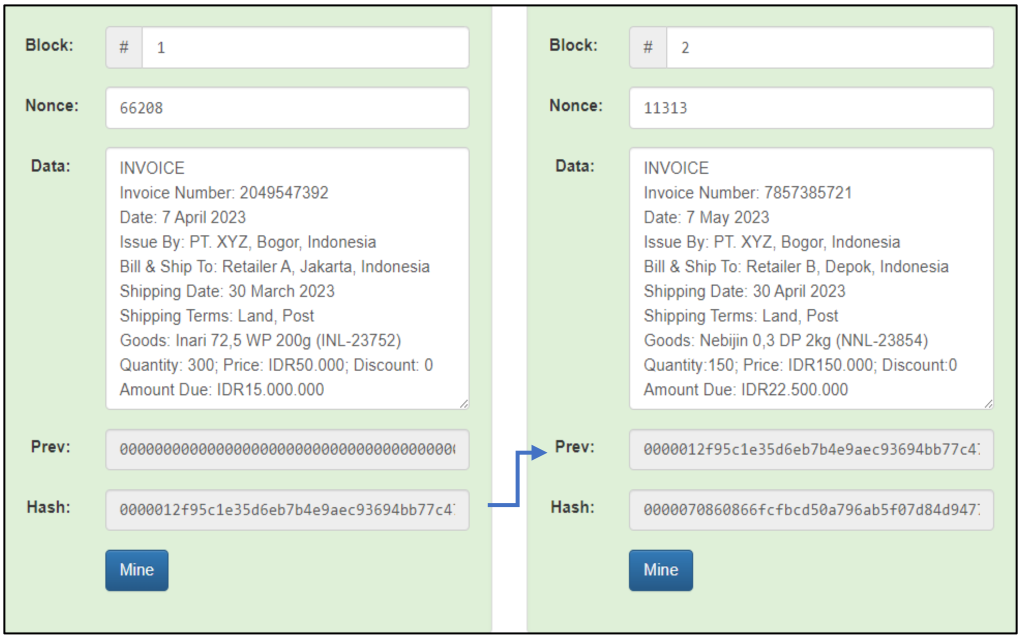

Figure 30 Company Invoice Simulation Results in Block 1 and Block 2

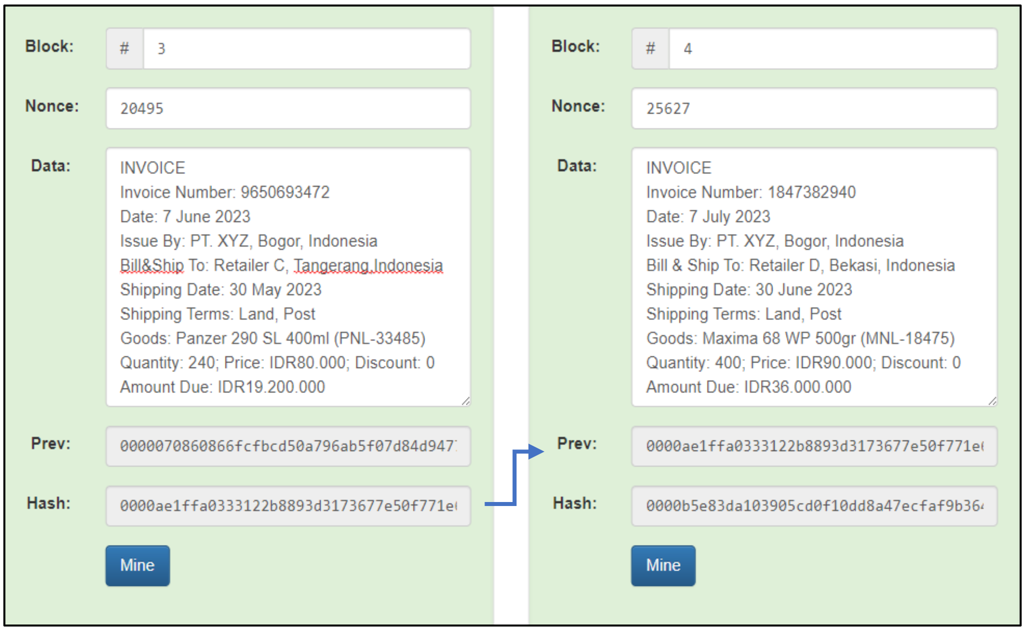

Figure 31 Company Invoice Simulation Results in Block 3 and Block 4

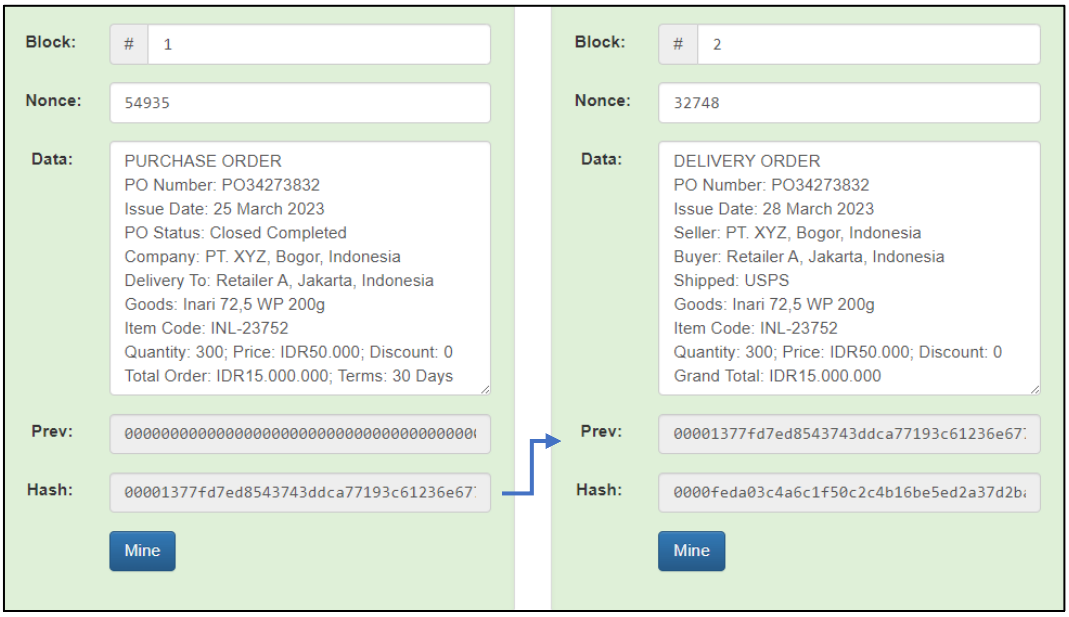

Figure 32 Simulation Results According to the Order of the Product Sales Process in Block 1 and Block 2

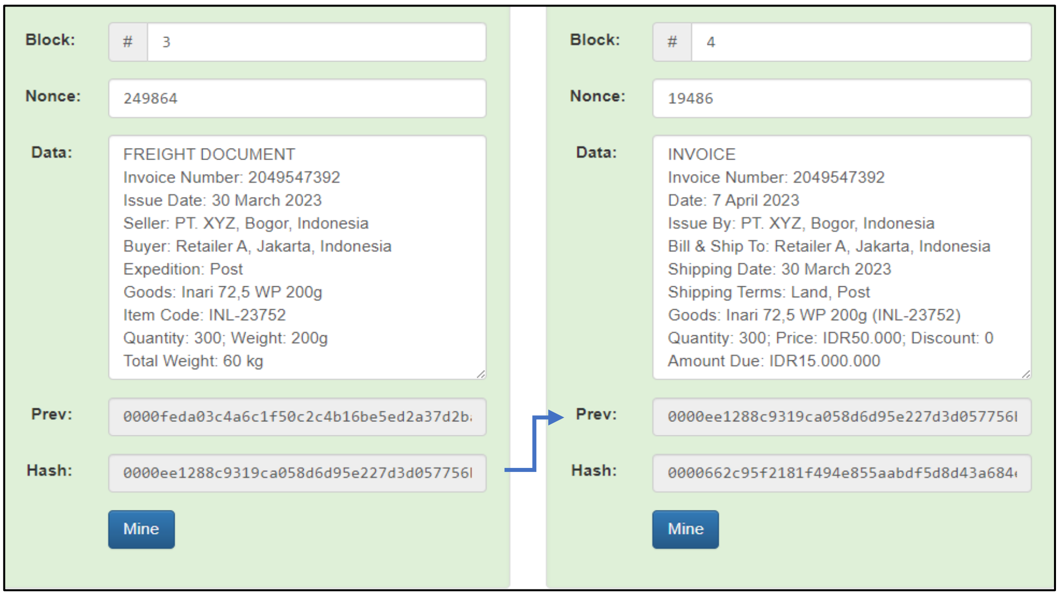

Figure 33 Simulation Results According to the Order of the Product Sales Process in Block 3 and Block 4

.

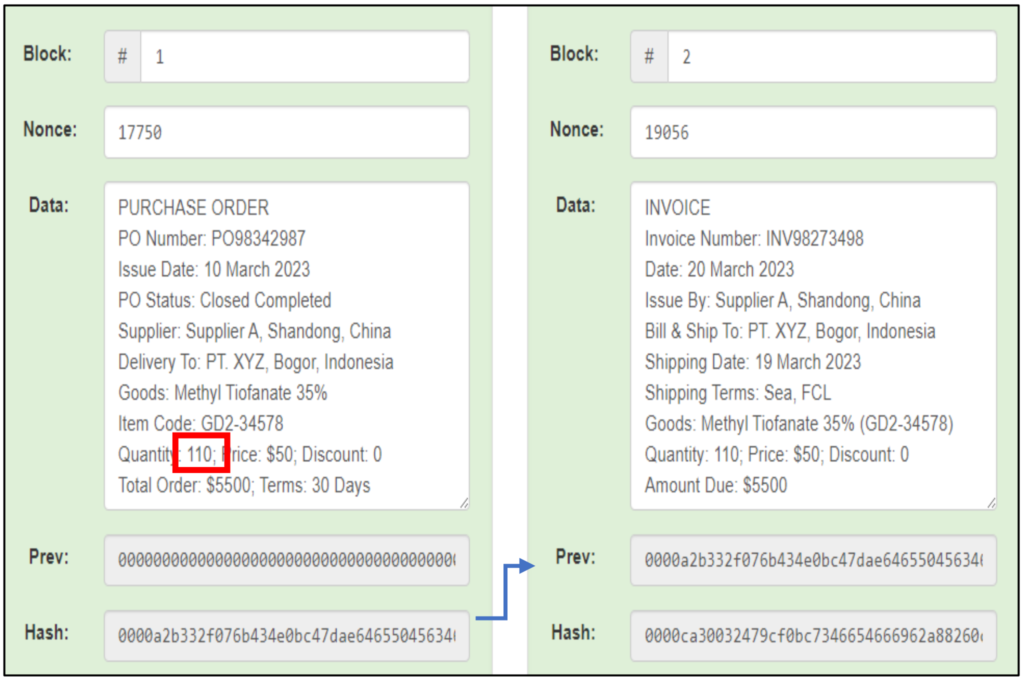

Figure 34 Saved Data Before Data Changes Occur

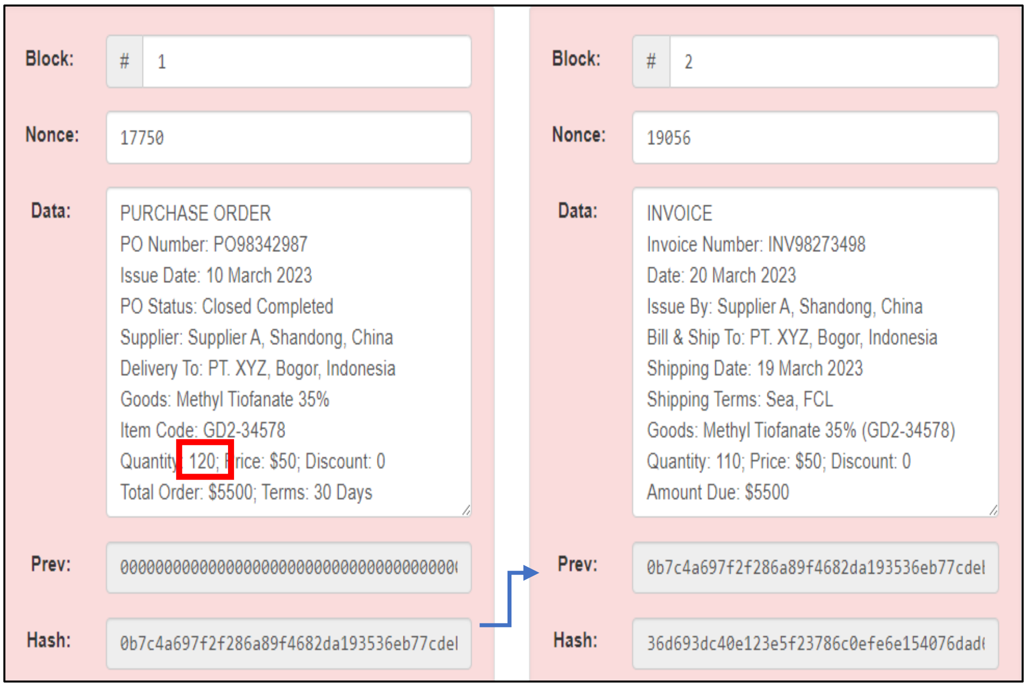

Figure 35 Data is saved after data changes are made

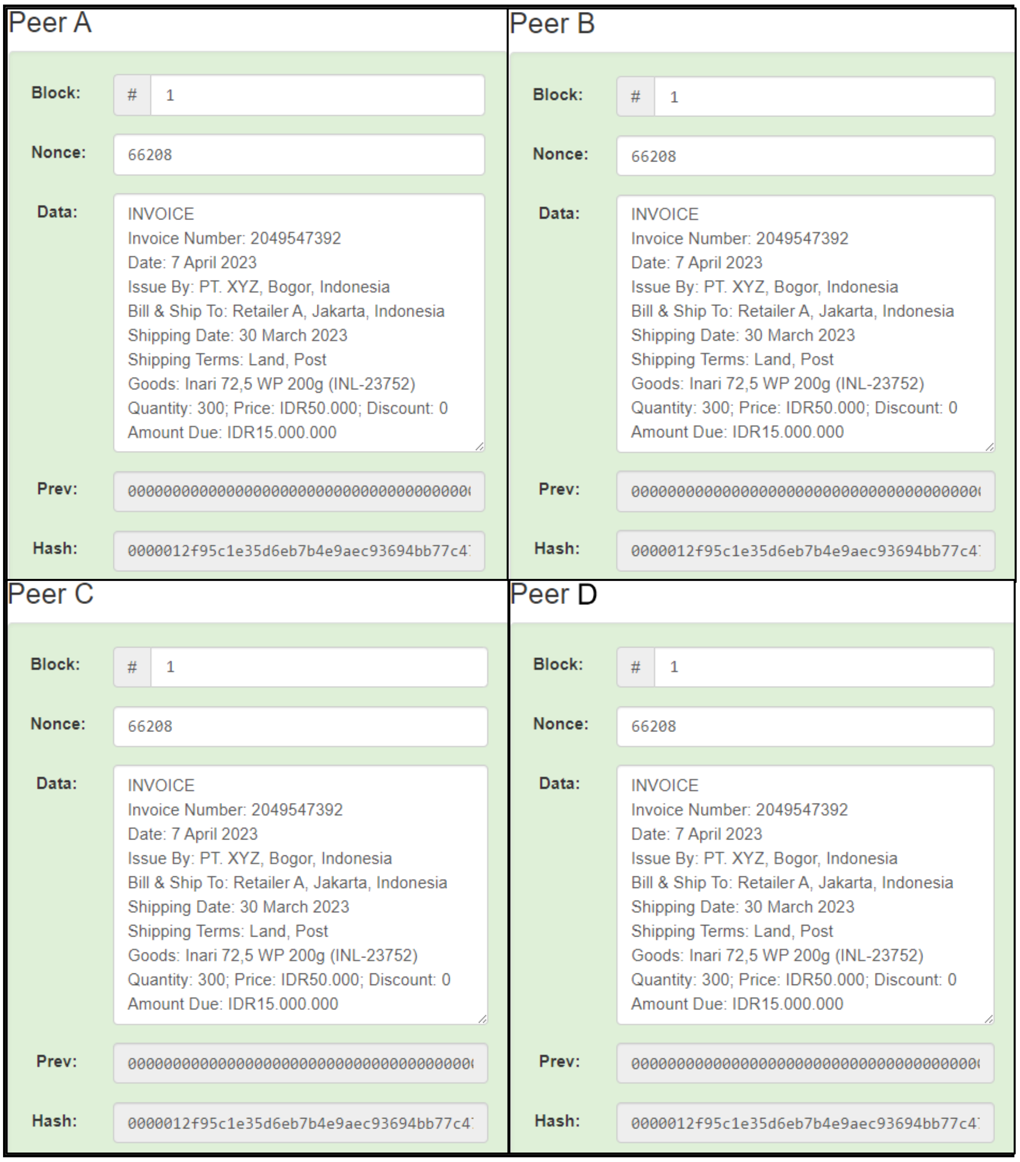

Figure 36 Data Storage Transparency among Supply Chain Actors

Data 1 : Data Assumptions in Contract Simulation (Raw Material Procurement Process)

Blok 1:

SUPPLIER AGREEMENT

(First Party)

PT. XYZ, Budi as Director

(Second Party)

China Supplier, Doni as Director

The Parties Here To Agree As Follows:

1. Definitions and Interpretation
2. Agreement to Supply
3. Term
4. Compensation and Payment

Blok 2:

SUPPLIER AGREEMENT

(First Party)

PT. XYZ, Budi as Director

(Second Party)

Japan Supplier, Mira as Director

The Parties Here To Agree As Follows:

1. Definitions and Interpretation
2. Agreement to Supply
3. Term
4. Compensation and Payment

Blok 3:

SUPPLIER AGREEMENT

(First Party)

PT. XYZ, Budi as Director

(Second Party)

Belgia Supplier, Natan as Director

The Parties Here To Agree As Follows:

1. Definitions and Interpretation
2. Agreement to Supply
3. Term
4. Compensation and Payment

Blok 4:

SUPPLIER AGREEMENT

(First Party)

PT. XYZ, Budi as Director

(Second Party)

Malaysia Supplier, Andi as Director

The Parties Here To Agree As Follows:

1. Definitions and Interpretation
2. Agreement to Supply
3. Term
4. Compensation and Payment

Data 2. Data Assumptions in Proforma Invoice Simulation (Raw Material Procurement Process)

Blok 1:
PROFORMA INVOICE
Invoice Number: INV – 2349459240
Issue Date: 01 March 2023
Seller: Supplier A, Shandong, China
Buyer: PT. XYZ, Bogor, Indonesia
Method of Dispatch: Sea
Type of Shipment: FCL
Method of Payment: 30% Deposit
Goods: Methyl Tiofanate 35%; Quantity: 100; Price: $50; Total: $5000

Blok 2:
PROFORMA INVOICE
Invoice Number: INV – 2345609784
Issue Date: 01 April 2023
Seller: Supplier B, Tokyo, Japan
Buyer: PT. XYZ, Bogor, Indonesia
Method of Dispatch: Sea
Type of Shipment: FCL
Method of Payment: 30% Deposit
Goods: Trisikazol 37.5%; Quantity: 100; Price: $60; Total: $6000

Blok 3:
PROFORMA INVOICE
Invoice Number: INV – 6908734124
Issue Date: 01 May 2023
Seller: Supplier C, Brabant, Belgium
Buyer: PT. XYZ, Bogor, Indonesia
Method of Dispatch: Sea
Type of Shipment: FCL
Method of Payment: 30% Deposit
Goods: Flusulfamide 0.3%; Quantity: 80; Price: $100; Total: $8000

Blok 4:
PROFORMA INVOICE
Invoice Number: INV – 0986678323
Issue Date: 01 June 2023
Seller: Supplier D, Kuala Lumpur, Malaysia

Buyer: PT.XYZ, Bogor, Indonesia
Method of Dispatch: Sea
Type of Shipment: FCL
Method of Payment: 30% Deposit
Goods: Mancozeb 80%; Quantity: 80; Price: $110; Total: $8800

Data 3.
Data Assumptions in Contract Simulation (Raw Material Procurement Process)

Blok 1:
PURCHASE ORDER
PO Number: PO98342987
Issue Date: 10 March 2023
PO Status: Closed Completed
Supplier: Supplier A, Shandong, China
Delivery To: PT. XYZ, Bogor, Indonesia
Goods: Methyl Tiofanate 35%
Item Code: GD2-34578
Quantity: 110; Price: $50; Discount: 0
Total Order: $5500; Terms: 30 Days

Blok 2:
PURCHASE ORDER
PO Number: PO45897234
Issue Date: 10 April 2023
PO Status: Closed Completed
Supplier: Supplier B, Tokyo, Japan
Delivery To: PT. XYZ, Bogor, Indonesia
Goods: Trisikazol 37.5%
Item Code: GD2-23498
Quantity: 120; Price: $60; Discount: 0
Total Order: $7200; Terms: 30 Days

Blok 3:
PURCHASE ORDER
PO Number: PO98743621
Issue Date: 10 May 2023
PO Status: Closed Completed
Supplier: Supplier C, Brabant, Belgium
Delivery To: PT. XYZ, Bogor, Indonesia
Goods: Flusulfamide 0.3%
Item Code: GD2-98473
Quantity: 90; Price: $100; Discount: 0
Total Order: $9000; Terms: 30 Days

Blok 4:
PURCHASE ORDER
PO Number: PO13847593
Issue Date: 10 June 2023
PO Status: Closed Completed
Supplier: Supplier D, Kuala Lumpur, Malaysia
Delivery To: PT. XYZ, Bogor, Indonesia
Goods: Mancozeb 80%
Item Code: GD2-17847
Quantity: 85; Price: $110; Discount: 0
Total Order: $9350; Terms: 30 Days

Data 4. Data Assumptions in Supplier Invoice Simulation (Raw Material Procurement Process)

Blok 1:
INVOICE
Invoice Number: INV98273498
Date: 20 March 2023
Issue By: Supplier A, Shandong, China
Bill & Ship To: PT. XYZ, Bogor, Indonesia
Shipping Date: 19 March 2023
Shipping Terms: Sea, FCL
Goods: Methyl Tiofanate 35% (GD2-34578)
Quantity: 110; Price: $50; Discount: 0
Amount Due: $5500

Blok 2:
INVOICE
Invoice Number: INV23958473
Date: 20 April 2023
Issue By: Supplier B, Tokyo, Japan
Bill & Ship To: PT. XYZ, Bogor, Indonesia
Shipping Date: 19 April 2023
Shipping Terms: Sea, FCL
Goods: Trisikazol 37.5% (GD2-23498)
Quantity: 120; Price: $60; Discount: 0
Amount Due: $7200

Blok 3:
INVOICE
Invoice Number: INV12128474
Date: 20 May 2023
Issue By: Supplier C, Brabant, Belgium
Bill & Ship To: PT. XYZ, Bogor, Indonesia
Shipping Date: 19 May 2023

Shipping Terms: Sea, FCL
Goods: Flusulfamide 0.3% (GD2-98473)
Quantity: 90; Price: $100; Discount: 0
Amount Due: $9000

Blok 4:
INVOICE
Invoice Number: INV03475483
Date: 20 June 2023
Issue By: Supplier D, Kuala Lumpur, Malaysia
Bill & Ship To: PT. XYZ, Bogor, Indonesia
Shipping Date: 19 June 2023
Shipping Terms: Sea, FCL
Goods: Mancozeb 80% (GD2-17847)
Quantity: 85; Price: $110; Discount: 0
Amount Due: $9350

Data 5. Data Assumptions in Payment Note Simulation (Raw Material Procurement Process)

Blok 1:
PAYMENT NOTE
Issue Date: 15 April 2023
Issue By: PT. XYZ, Bogor, Indonesia
To: Supplier A, Shandong, China
Bill Ref.: New Ref/ Bill Fdg/04b23
Bill Date: 20 March 2023
Payment Mode: Inter Bank Transfer
A/c No.: 0043953823024823
IFSC: IOBA0003854238
Nett Amount: $5500

Blok 2:
PAYMENT NOTE
Issue Date: 15 May 2023
Issue By: PT. XYZ, Bogor, Indonesia
To: Supplier B, Tokyo, Japan
Bill Ref.: New Ref/ Bill Gbh/05h238
Bill Date: 20 April 2023
Payment Mode: Inter Bank Transfer
A/c No.: 0004957498732092
IFSC: IOBA0002364582
Nett Amount: $7200

Blok 3:

PAYMENT NOTE
Issue Date: 15 June 2023
Issue By: PT. XYZ, Bogor, Indonesia
To: Supplier C, Brabant, Belgium
Bill Ref.: New Ref/ Bill Jns/08s328
Bill Date: 20 May 2023
Payment Mode: Inter Bank Transfer
A/c No.: 0001298985482347
IFSC: IOBA0009284548
Nett Amount: $9000

Blok 4:
PAYMENT NOTE
Issue Date: 15 July 2023
Issue By: PT. XYZ, Bogor, Indonesia
To: Supplier D, Kuala Lumpur, Malaysia
Bill Ref.: New Ref/ Bill Kjg/06t847
Bill Date: 20 June 2023
Payment Mode: Inter Bank Transfer
A/c No.: 0008274695720183
IFSC: IOBA0004238354
Nett Amount: $9350

Data 6. Data Assumptions in Buyer Purchase Order (PO) Simulation (Product Sales Process)

Blok 1:
PURCHASE ORDER
PO Number: PO34273832
Issue Date: 25 March 2023
PO Status: Closed Completed
Company: PT. XYZ, Bogor, Indonesia
Delivery To: Retailer A, Jakarta, Indonesia
Goods: Inari 72,5 WP 200g
Item Code: INL-23752
Quantity: 300; Price: IDR50.000; Discount: 0
Total Order: IDR15.000.000; Terms: 30 Days

Blok 2:
PURCHASE ORDER
PO Number: PO284659270
Issue Date: 25 April 2023
PO Status: Closed Completed
Company: PT. XYZ, Bogor, Indonesia
Delivery To: Retailer B, Depok, Indonesia
Goods: Nebijin 0,3 DP 2kg

Item Code: NNL-23854
Quantity: 150; Price: IDR150.000; Discount: 0
Total Order: IDR22.500.000; Terms: 30 Days

Blok 3:
PURCHASE ORDER
PO Number: PO85729473
Issue Date: 25 May 2023
PO Status: Closed Completed
Company: PT. XYZ, Bogor, Indonesia
Delivery To: Retailer C, Tangerang, Indonesia
Goods: Panzer 290 SL 400ml
Item Code: PNL-33485
Quantity: 240; Price: IDR80.000; Discount: 0
Total Order: IDR19.200.000; Terms: 30 Days

Blok 4:
PURCHASE ORDER
PO Number: PO54738533
Issue Date: 25 June 2023
PO Status: Closed Completed
Company: PT. XYZ, Bogor, Indonesia
Delivery To: Retailer D, Bekasi, Indonesia
Goods: Maxima 68 WP 500gr
Item Code: MNL-18475
Quantity: 400; Price: IDR90.000; Discount: 0
Total Order: IDR36.000.000; Terms: 30 Days

Data 7. Data Assumptions in Delivery Order (DO) Simulation (Product Sales Process)

Blok 1:
DELIVERY ORDER
PO Number: PO34273832
Issue Date: 28 March 2023
Seller: PT. XYZ, Bogor, Indonesia
Buyer: Retailer A, Jakarta, Indonesia
Shipped: USPS
Goods: Inari 72,5 WP 200g
Item Code: INL-23752
Quantity: 300; Price: IDR50.000; Discount: 0
Grand Total: IDR15.000.000

Blok 2:

DELIVERY ORDER
PO Number: PO284659270
Issue Date: 28 April 2023
Seller: PT. XYZ, Bogor, Indonesia
Buyer: Retailer B, Depok, Indonesia
Shipped: USPS
Goods: Nebijin 0,3 DP 2kg
Item Code: NNL-23854
Quantity:150; Price: IDR150.000; Discount:0
Grand Total: IDR22.500.000

Blok 3:
DELIVERY ORDER
PO Number: PO85729473
Issue Date: 28 May 2023
Seller: PT. XYZ, Bogor, Indonesia
Buyer: Retailer C, Tangerang, Indonesia
Shipped: USPS
Goods: Panzer 290 SL 400ml
Item Code: PNL-33485
Quantity: 240; Price: IDR80.000; Discount: 0
Grand Total: IDR19.200.000

Blok 4:
DELIVERY ORDER
PO Number: PO54738533
Issue Date: 28 June 2023
Seller: PT. XYZ, Bogor, Indonesia
Buyer: Retailer D, Bekasi, Indonesia
Shipped: USPS
Goods: Maxima 68 WP 500gr
Item Code: MNL-18475
Quantity: 400; Price: IDR90.000; Discount: 0
Grand Total: IDR36.000.000

Data 8. Data Assumptions in Travel Document Simulation (Product Sales Process)

Blok 1:
FREIGHT DOCUMENT
Invoice Number: 2049547392
Issue Date: 30 March 2023
Seller: PT. XYZ, Bogor, Indonesia
Buyer: Retailer A, Jakarta, Indonesia
Expedition: Post
Goods: Inari 72,5 WP 200g

Item Code: INL-23752
Quantity: 300; Weight: 200g
Total Weight: 60 kg

Blok 2:
FREIGHT DOCUMENT
Invoice Number: 7857385721
Issue Date: 30 April 2023
Seller: PT. XYZ, Bogor, Indonesia
Buyer: Retailer B, Depok, Indonesia
Expedition: Post
Goods: Nebijin 0,3 DP 2kg
Item Code: NNL-23854
Quantity:150; Weight: 2 kg
Total Weight: 300 kg

Blok 3:
FREIGHT DOCUMENT
Invoice Number: 9650693472
Issue Date: 30 May 2023
Seller: PT. XYZ, Bogor, Indonesia
Buyer: Retailer C, Tangerang, Indonesia
Expedition: Post
Goods: Panzer 290 SL 400ml
Item Code: PNL-33485
Quantity: 240; Weight: 350 g
Total Weight: 84 kg

Blok 4:
FREIGHT DOCUMENT
Invoice Number: 1847382940
Issue Date: 30 June 2023
Seller: PT. XYZ, Bogor, Indonesia
Buyer: Retailer D, Bekasi, Indonesia
Expedition: Post
Goods: Maxima 68 WP 500gr
Item Code: MNL-18475
Quantity: 400; Weight: 500 gr
Total Weight: 200 kg

Data 9.
Data Assumptions in Company Invoice Simulation (Product Sales Process)

Blok 1:
INVOICE
Invoice Number: 2049547392
Date: 7 April 2023
Issue By: PT. XYZ, Bogor, Indonesia

Bill & Ship To: Retailer A, Jakarta, Indonesia
Shipping Date: 30 March 2023
Shipping Terms: Land, Post
Goods: Inari 72,5 WP 200g (INL-23752)
Quantity: 300; Price: IDR50.000; Discount: 0
Amount Due: IDR15.000.000

Blok 2:
INVOICE
Invoice Number: 7857385721
Date: 7 May 2023
Issue By: PT. XYZ, Bogor, Indonesia
Bill & Ship To: Retailer B, Depok, Indonesia
Shipping Date: 30 April 2023
Shipping Terms: Land, Post
Goods: Nebijin 0,3 DP 2kg (NNL-23854)
Quantity:150; Price: IDR150.000; Discount:0
Amount Due: IDR22.500.000

Blok 3:
INVOICE
Invoice Number: 9650693472
Date: 7 June 2023
Issue By: PT. XYZ, Bogor, Indonesia
Bill&Ship To: Retailer C, Tangerang,Indonesia
Shipping Date: 30 May 2023
Shipping Terms: Land, Post
Goods: Panzer 290 SL 400ml (PNL-33485)
Quantity: 240; Price: IDR80.000; Discount: 0
Amount Due: IDR19.200.000

Blok 4:
INVOICE
Invoice Number: 1847382940
Date: 7 July 2023
Issue By: PT. XYZ, Bogor, Indonesia
Bill & Ship To: Retailer D, Bekasi, Indonesia
Shipping Date: 30 June 2023
Shipping Terms: Land, Post
Goods: Maxima 68 WP 500gr (MNL-18475)
Quantity: 400; Price: IDR90.000; Discount: 0
Amount Due: IDR36.000.000

Data 10. Data from discussions with resource persons

Person 1

- The supply chain network structure needs to be considered, there needs to be a raw material warehouse if there is a raw material warehouse in the supply chain. If you don't want to use both, you can just write to the company.
- The number of sources can be added to complete the discussion results so that they become more accurate and objectivity can increase.
- Analysis of system requirements is paid attention to by the actors, pay attention to the terms used in the supply chain so as not to use the terms incorrectly.
- Analysis of blockchain needs in the question and answer table needs to be sharpened and answered in detail so that the explanation is clear and does not raise further questions that cause confusion.
- Re-examine the theory used so that there are no mistakes in using existing theories.
- If secondary data is used, the company name can be disguised.
- When determining the subprocess, several changes occur in some subprocesses.

Person 2

- Pay attention to the results of the analysis in the sustainability aspect. The aspect in question must be clarified as to what is meant by sustainability. If sustainability to data must be mentioned.
- Determination of subprocesses must be clear and objective. There needs to be experts who can help in making considerations in it.
- The company name can only be disguised. Because the data used is not primary data.
- Input, process and output need to be explained in order to provide a comprehensive picture.

Person 3

- The simulation design must be sequential and interconnected. The relationship between them in the design process is also explained.
- Making use case diagrams and activity diagrams must be clear. According to the purpose. The format for making the two diagrams must also be in accordance with the commonly used format.
- Determining subprocesses needs to be added to the number of aspects used. With the aim that the results of subprocess determination can be even better. Improvements to subprocess determination results.
- The simulation results must be clear about the data used in each process. The data needs to be assumed well and can represent the document.
- Explained in detail the simulation results related to hash and previous hash in the image and their function clearly. Then the aspect analysis must also be deep.

Person 4

- The use of titles must be appropriate. Because it uses secondary data and only explores the system that is observed, not the company, it needs to be turned into a case study. With the aim of focusing on the system being observed, not a particular company that needs to be known in detail.
- When determining subprocesses, an explanation needs to be given regarding marking in the table. The data exchange aspect is marked with a cross because the data is confidential. Such as the materials used to produce pesticides. Then the company registration data is also confidential and cannot be known to other parties because it contains the organizational structure and so on.
- Pay attention to the use of company names because the data obtained is not data taken directly from the company concerned, so the company name does not need to be mentioned in the report.
- The pesticide supply chain needs further attention to ensure that it is more in line with the pesticide supply chain in general. Then the actors in the pesticide supply chain can also be re-examined whether they are appropriate.
